# Disorder-to-order active site capping regulates the rate-limiting step of the inositol pathway

**DOI:** 10.1073/pnas.2400912121

**Published:** 2024-08-15

**Authors:** Toni K. Träger, Fotis L. Kyrilis, Farzad Hamdi, Christian Tüting, Marie Alfes, Tommy Hofmann, Carla Schmidt, Panagiotis L. Kastritis

**Affiliations:** ^a^Faculty of Natural Sciences I, Institute of Biochemistry and Biotechnology, Martin Luther University Halle-Wittenberg, Halle/Saale 06120, Germany; ^b^Biozentrum, Martin Luther University Halle-Wittenberg, Halle/Saale 06120, Germany; ^c^Institute of Chemical Biology, National Hellenic Research Foundation, Athens 11635, Greece; ^d^Interdisciplinary Research Center HALOmem, Charles Tanford Protein Center, Martin Luther University Halle-Wittenberg, Halle/Saale 06120, Germany; ^e^Biologics Analytical R&D, AbbVie Deutschland GmbH & Co. KG, Ludwigshafen 67061, Germany; ^f^Impfstoffwerk Dessau-Tornau Biologika, Dessau-Roßlau 06861, Germany; ^g^Department of Chemistry–Biochemistry, Johannes Gutenberg University Mainz, Mainz 55128, Germany

**Keywords:** endogenous, cryo-EM, induced fit, inositol metabolism, conformational selection

## Abstract

Myo-inositol-1-phosphate synthase (MIPS) catalyzes the NAD^+^-dependent isomerization of glucose-6-phosphate (G6P) into inositol-1-phosphate (IMP), controlling the rate-limiting step of the inositol pathway. Previous structural studies focused on the detailed molecular mechanism, neglecting large-scale conformational changes that drive the function of this 240 kDa homotetrameric complex. In this study, we identified the active, endogenous MIPS in cell extracts from the thermophilic fungus *Thermochaetoides thermophila*. By resolving the native structure at 2.48 Å (FSC = 0.143), we revealed a fully populated active site. Utilizing 3D variability analysis, we uncovered conformational states of MIPS, enabling us to directly visualize an order-to-disorder transition at its catalytic center. An acyclic intermediate of G6P occupied the active site in two out of the three conformational states, indicating a catalytic mechanism where electrostatic stabilization of high-energy intermediates plays a crucial role. Examination of all isomerases with known structures revealed similar fluctuations in secondary structure within their active sites. Based on these findings, we established a conformational selection model that governs substrate binding and eventually inositol availability. In particular, the ground state of MIPS demonstrates structural configurations regardless of substrate binding, a pattern observed across various isomerases. These findings contribute to the understanding of MIPS structure-based function, serving as a template for future studies targeting regulation and potential therapeutic applications.

## Significance

Myo-inositol-1-phosphate synthase (MIPS) controls inositol availability, a second messenger in signaling. Previous structural studies elucidated MIPS catalysis, but the broader conformational changes that drive function remain elusive, especially in the enzyme’s endogenous state. This study elucidated intricate mechanisms governing the native enzyme activity. MIPS, directly derived from the fungus *Thermochaetoides thermophila* with minimal isolation steps, exhibits disorder-to-order transitions localized at its catalytic site which is occupied by an acyclic reaction intermediate. This work thereby illuminates structural, molecular, and catalytic mechanisms behind the inositol pathway, and their conservation across isomerases, and paves the way for therapeutic interventions, especially considering the enzyme’s significant role in cellular viability and function.

Inositol-derived compounds, namely polyphosphorylated inositol analogues (IPs) as well as phosphatidylinositol phosphates (PIPs), play major roles in cellular signaling (1–3), membrane formation (4, 5), DNA repair (6), and energy metabolism (7, 8), making them of high importance for structural characterization and clinical research (9–11). The initial steps of inositol biosynthesis are preserved in all kingdoms (5). In the first step of the reaction, glucose-6-phosphate (G6P) is converted to myo-inositol-1-phosphate (IMP) by myo-inositol-1-phosphate synthase (MIPS) (12) and subsequently dephosphorylated (*SI Appendix*, Fig. S1*A*). These compounds serve as crucial components in cellular processes and are essential for the proper function of cellular systems (13).

So far, MIPS has been structurally resolved in prokaryotic and eukaryotic organisms (14–17), leading to an analysis of the underlying molecular mechanism. In vitro studies of the archaeal homologue (18) revealed 5 distinct catalytic steps (*SI Appendix*, Fig. S1*A*) that are carried out by a network of lysine residues (15, 19–21): i) G6P, initially bound in its pyranose form, is protonated by the catalytic residues, leading to ring opening; ii) the open chain G6P is oxidized by NAD^+^, resulting in the formation of a keto group at the C5 carbon; iii) enolization of the C5─C6 bond results in the iv) nucleophilic attack of the C1 aldehyde group and ring closure; and v) finally, NAD^+^ is regenerated through the reduction of the C5 ketone group. Previous crystallographic studies indirectly observed the unfolding of a helical domain at the entrance of the active site of MIPS (14, 22), postulating an induced fit mechanism as this step is directly involved in the catalytic mechanism, through the contribution of two catalytic lysine residues. The function of the disorder-to-order transition of this element and its role in the catalytic cycle remain elusive, failing to relate structure to protein function.

Although crucial for the understanding of MIPS function, these mechanistic observations were studied in the context of isolated in vitro preparations. It has been shown that phosphorylation of residues adjacent to the active site drastically impacts MIPS activity (23–25) while changes in MIPS abundance are proposed to be the major determinant of the inositol pathway in vivo (26). Posttranslational modifications (PTMs) as well as cellular interactors, directly attenuating MIPS function (27), are therefore removed from the structural analysis. To overcome the traditional limitations posed by purification schemes and protein crystallography approaches, cryogenic electron microscopy (cryo-EM) can serve as a powerful tool to investigate conformational changes, protein assembly, and protein interactions directly in a heterogeneous cell lysate (28) (*SI Appendix*, Fig. S1*B*).

We therefore describe an approach to directly identify active MIPS complex across fractions (size-based fractionation) of cell extracts. The complex was resolved at 2.48 Å (FSC=0.143), revealing clear domain organization and high-resolution information of the endogenous state, indicating a fully populated active site. Using molecular docking and refinement analyses, we determined the primary binding interfaces, comparing energetic forces of eukaryotic and prokaryotic MIPS assembly. 3D variability analysis enabled visualization of changes in the folding state of a helical domain which forms part of the active site, therefore regulating access while analysis of all isomerases exhibits similar flexibility of active site-embedded secondary structures. We consequently establish a quantitative model for the catalytic cycle of MIPS, a reaction driven by a disorder-to-order transition, and elucidate endogenous conformational states of this metabolic complex.

## Results

### Myo-Inositol-Phosphate Synthase (MIPS) from Endogenous Cell Extracts Exhibits Native-State Adaptations.

**Fig. 1.**
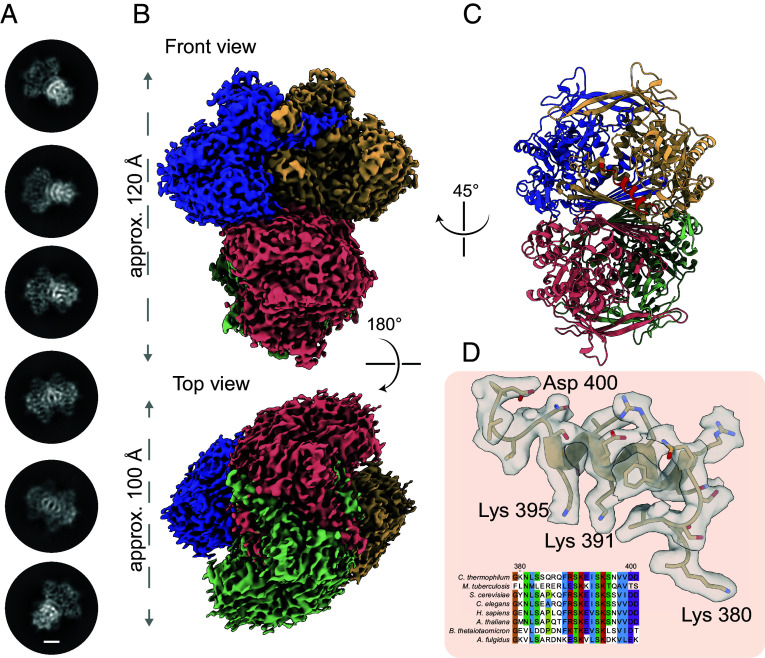
Biochemical analysis of the endogenous myo-inositol-phosphate synthase. (*A*) Representative 2D classes of MIPS are shown. The scale bar indicates 2.5 nm. (*B*) Density map of endogenous MIPS reconstructed at 2.48 Å; individual subunits of the tetrameric complex are colored (contour level at 0.2). (*C*) Final model of the endogenous MIPS reveals higher-order organization of the 240 kDa tetrameric complex. Helical domain at the entry of the active site marked in red. (*D*) The helical domain between residues Lys 380 and Asp 400 is highly conserved across a plethora of organisms (insert). Helical morphology and side chain densities are clearly visible in the final reconstruction (contour level at 0.2).

We utilized a minimal purification approach to capture the endogenous MIPS, preserving elements of cellular organization, and rendering the sample suitable for structural characterization (28) (*SI Appendix*, Fig. S1*B*). This was achieved by separation of crude cell extract, derived from the thermophilic filamentous fungus *Thermochaetoides thermophila*, using two centrifugation steps (*SI Appendix*, Fig. S2) followed by size exclusion chromatography (SEC) (*SI Appendix*, Fig. S3*A*). We subsequently employed label-free mass spectrometry (MS) to identify the retention time of fungal MIPS within the fractionated, soluble proteome (*SI Appendix*, Fig. S3*B*). To probe the identified fractions for their capacity to convert G6P to IMP, we deployed an activity assay to verify the native state of MIPS by detecting phosphate release at 820 nm (*Materials and Methods*). MS as well as activity measurements complementary identified MIPS in consecutive elution fractions (fractions 30 to 40) where both data correlated in terms of quantitative measurements and formed overlapping profiles (*SI Appendix*, Fig. S3*B*). Focused cryo-EM analysis of the vitrified fraction 35 (*SI Appendix*, Fig. S3*C*), chosen based on the highest activity and MS abundance (*SI Appendix*, Fig. S3*B*), revealed MIPS (Fig. 1*A*) as well as 4 more prominent signatures belonging to the fungal glutamine synthetase, the pyruvate kinase, and two more unknown complexes (*SI Appendix*, Fig. S3 *C* and *D*)—the identified belonging to a metabolic community previously predicted by network biology (28) and seen in the analyzed fraction (*SI Appendix*, Fig. S4 *A* and *B*).

After sample optimization, data acquisition, and initial image analysis (*SI Appendix*, Fig. S5), we resolved the MIPS initially at 3.0 Å (FSC = 0.143). Extensive image analysis was performed, utilizing reference-based motion correction (*Materials and Methods*), resulting in a final reconstruction at 2.48 Å (FSC = 0.143) (*SI Appendix,* Table S1 and Fig. S6 *A–E*). The fungal MIPS from the thermophile, as its mesophilic counterparts, forms a homotetramer, with dimensions of 120 Å by 100 Å (Fig. 1*B*), highlighting that the overall stoichiometry is conserved. In the final reconstruction of the endogenous complex, we were able to resolve residues 40 to 552, which overall reveal a high conservation among all structurally determined MIPS orthologues. The N-terminal region, notable for its low complexity (*SI Appendix*, Fig. S7*A*) could not be resolved, most likely due to its flexible nature, but is also predicted to be present in the human MIPS sequence (*SI Appendix*, Fig. S7*B*). Intriguingly, this characteristic of a disordered N terminus is also observed in prokaryotic homologues, whereas in higher eukaryotes, this feature is shifted to the C terminus or is entirely absent in plants (*SI Appendix*, Fig. S8 *A* and *B*).

Overall, the higher-order organization of fungal MIPS is in line with previously determined orthologous structures (Fig. 1*C*) (29), featuring an extensive β-sheet network at a horizontal dimerization interface, a highly conserved NAD^+^ binding domain and an extensive vertical central domain connecting the monomers (*SI Appendix*, Fig. S9 *A* and *B*). In more detail, the horizontal interface is primarily stabilized by hydrophobic interactions and an extensive hydrogen bond network (*SI Appendix*, Fig. S10*A*), while the vertical interface features a large area of buried residues, stabilized by electrostatic interactions (*SI Appendix*, Fig. S10*A*). Across all analyzed interfaces, including both prokaryotic and eukaryotic structures of MIPS (*SI Appendix*, Tables S2 and S3), it was discerned that the vertical interfaces manifest a pronounced disparity in buried surface area, rendering them more energetically advantageous. We observed notable progression in electrostatic forces with *T. thermophila* serving as the pivotal bridge linking prokaryotic MIPS to its higher eukaryotic counterparts (*SI Appendix*, Fig. S10*B*). Compared to other eukaryotic structures (30), the thermophilic model is distinguished by a) conformational changes of the central domain (*SI Appendix*, Fig. S9*A*) and b) several instances of shortened loops (*SI Appendix*, Fig. S9*B*), overall contributing to a more compact structure. Residues 380 to 400, guarding the entry to the active site appeared to be in a folded state, showing clear α-helical morphology (Fig. 1*D*). To analyze α-helix propensity, we calculated per residue α-helix likelihood by comparing the fungal MIPS to its prokaryotic homologue (18) (*SI Appendix*, Fig. S11). A decrease between residues 388 and 398 was observed, possibly forming a site for α-helix unfolding, while the prokaryotic sequence generally showed a higher propensity for α-helix formation.

### Revisiting the Active Site Structure of MIPS.

**Fig. 2.**
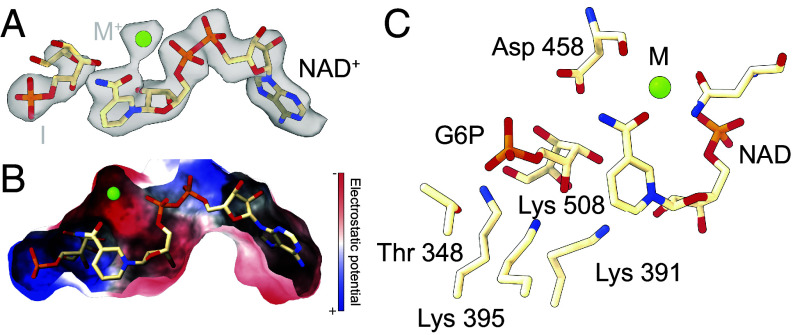
Reconstruction of native fungal MIPS reveals a fully populated active site. (*A*) The density map and the model of endogenous MIPS reveal a populated active site. NAD could be unambiguously fitted while an acyclic intermediate (I) and the representative ion were refined, based on identified cryo-EM densities, homologous active sites, and previous crystallographic placement (contour level at 0.075). (*B*) The electrostatic surface potential map of the active site was calculated, revealing highly charged regions surrounding the phosphate groups of NAD and G6P. (*C*) Residues that are in direct interaction with the isomerization reaction are displayed. Highly conserved residues Lys 391 and Lys 395, donated by the α-helical domain at the entry of the active site, facilitate G6P isomerization with the assistance of Lys 508.

The endogenous structure of the fungal MIPS displays a fully populated active site, allowing for the unambiguous placement of the cofactor NAD (Fig. 2*A*). In the asymmetric reconstruction of the complex, we observed persistent densities for NAD, illustrating that NAD occupies all four active sites simultaneously—i.e., MIPS is endogenously NAD saturated. While higher eukaryotes are readily activated by NH_4_^+^, archaic orthologues of MIPS have been characterized as class 2 aldolases, utilizing a divalent cation for increased activity (31). We observed a prominent density in line with previously characterized ion positions (*SI Appendix*, Fig. S12 *A* and *B*). The ion is complexed by a network of charged residues, most notably Asn 105, multiple structural waters as well as NAD headgroup and phosphate backbone interactions (between 2.4 to 3.2 Å), that are oriented to favor metal binding (*SI Appendix*, Fig. S12*C*). The coordination lengths presented would be unlikely for divalent- and more in line with monovalent cations, with potassium being the most likely option, displaying generally larger mean binding distances compared to sodium (32). A secondary ion responsible for direct catalytic interaction, as proposed previously (33), could not be identified. The location and spacing of this ion suggest that it is unlikely to play a direct role in catalysis, as seen in archaea. However, it remains part of a hydrogen bonding network that extends throughout the entire active site. Conducting a comparative analysis of NAD positioning across the active site cavities by considering the influence of metal ion binding we observed the nicotinamide headgroup to be stabilized upon ion binding (*SI Appendix*, Fig. S13*A*).

To determine the substrate binding mode, evaluating the positioning of the open or closed chain product, we calculated per residue Q-scores for the substrates (*SI Appendix*, Fig. S13*B*) (34). Evidently, we observed a clear indication for the existence of an acyclic substrate, e.g., G6P in its pyranose form, that provides the most accurate representation of the experimental observations. This model resembles the structure found in archaeal MIPS as well as the inhibitor-bound eukaryotic structure (14, 15), notably featuring a more compact distance of 3.4 Å between the C1 and C6 carbon atoms.

This placement is in agreement with the density patterns revealed through our analysis. A comprehensive examination of the electrostatic surface potential map unveiled consecutive surfaces in the active site with alternate charge (Fig. 2*B*), supplying charged binding domains that are pivotal for the interaction with the phosphate groups of G6P and NAD. Furthermore, these binding pockets encompass a substantial region that is primed for the interaction with a cation. In this configuration NAD is electrostatically positioned within the active site of MIPS, further stabilized by the electrostatic interactions with the cation. Such interactions are extremely stable, and therefore, NAD is unlikely to dissociate during catalysis. To evaluate NAD dissociation, we analyzed PDB-deposited structures (35) of eukaryotic and prokaryotic MIPS and could not establish any link between NAD binding and active site conformation changes (*SI Appendix*, Tables S2 and S3), supporting our hypothesis that NAD is tightly bound within the active site and does not dissociate during reaction cycles in the active endogenous purification (*SI Appendix*, Fig. S3 and Table S4). This hypothesis is also substantiated by the observation that NAD does not dissociate during biochemical treatment of the cell extract and is present in our reconstructions with full occupancy. The positioning of active site residues is in line with the previously described mechanisms and mutational analysis (14, 15) positioning Lys 391, 395, and 508 in juxtaposition for facilitating G6P isomerization (Fig. 2*C*).

### Conformational Space.

**Fig. 3.**
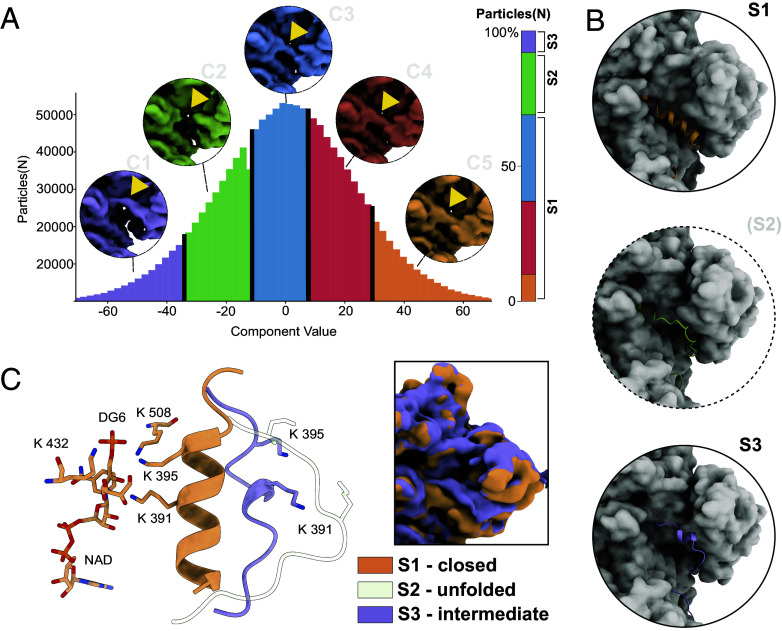
Exploring the conformational space of MIPS. (*A*) 3D variability analysis of MIPS reveals conformational heterogeneity at the active site, allowing the reconstruction of distinct structural substates (Clusters C1 to C5). Position of the α-helical domain between residues 380 and 400 marked (yellow arrow). A quantitative assessment of substates can be performed by assigning single particle images to three distinct substates, S1 (folded helical domain), S2 (unfolded helical domain), and S3 (alternative conformation). (*B*) Model of MIPS was refined in the reconstructed Coulomb potential map derived from the single particles that were clustered in (*A*). The reconstructed volumes as well as the α-helical domain are shown in cartoon representation. A critical conformational selection mechanism that is mediated by a disorder-to-order transition is hinted by deriving maps with absent and present helical densities, as well as 3D maps with α-helical occupancy between those extremes. (*C*) Five conserved lysine residues (391, 395, 432, and 508 are the *T. thermophila* counterparts) have been suggested to play a crucial role in MIPS catalysis. In the NAD-bound state (S1), glucose-6 phosphate is coordinated by Lys 391, 395, 432, and 508, forming a H–H bond interface. All conformations are present with a fully populated active site and therefore regulate catalysis across all identified steps. For the S3 state specifically, an alternate hypothesis is that it may represent an inhibited state, that is manifested due to a conformational rotation of the MIPS monomer (Insert).

Cryo-EM has seen significant advancements, enabling modern image analysis algorithms to explore the conformational dynamics of proteins encased in vitreous ice. Here, we employed 3D variability analysis (3DVA) (36), to delve into the molecular motion and heterogeneity of endogenous MIPS. To investigate the conformational change occurring in the ground state of MIPS, a total of 333,149 particles were analyzed, using three variability components at a filter resolution of 5 Å. Notably, one of the three variability components displayed the highest variance at the helical domain shielding the active site (residues 385 to 400) and was therefore chosen for further analysis (*SI Appendix*, Fig. S5). The particles were clustered in 5 groups, indicating a continuous change in the helical domain (Fig. 3*A*). From the reconstructed volumes across these clusters, we identified 3 conformational states (Fig. 3*B*). State 1 (S1) is characterized by a folded helical domain (23%), whereas this domain is absent in state 2 (S2) (69%), possibly due to unfolding, and backfolded in state 3 (8%). To investigate the alternative conformation, observed in states 2 (S2) and 3 (S3), we used the corresponding particles for de novo reconstruction (*SI Appendix*, Figs. S14 *A–E* and S15 *A–E*). The helical domain is shifted approx. 8 Å, completely exposing the active site in this backfolded conformation (*SI Appendix*, Fig. S16 *A–C*). We additionally observed an asymmetric distribution of the helical domain across the four active sites (*SI Appendix*, Fig. S16*A*), that must be linked to different active site states. To elucidate substrate-specific conformational change (*SI Appendix*, Fig. S17), we examined the active site substrate population across the three states. Densities corresponding to NAD and the previously described ion are visible in all but the acyclic reaction intermediate is visible in states 1 and 2 (but not 3; back-folded). Based on these results we postulate a conformational selection mechanism (37) for MIPS catalysis involving a disorder-to-order transition, gating the active site. Lys 391 and 395 are essential for the conversion of G6P, serving as proton donors and acceptors for intermediate reaction steps as seen in S2 (Fig. 3*C*). IMP release requires the unfolding of the helical domain between residues 385 to 405 as seen in state 2, depriving the active site of the catalytic lysine residues. State 3 represents an alternative binding mode for this domain, possibly serving as an intermediate between states 1 and 2 or as an inhibited form of MIPS, displaying additional domain rotation (Fig. 3*C*).

## Discussion

The understanding of the molecular architecture of MIPS is fundamental not only for the comprehension of inositol biosynthesis but also for the broader picture of cellular signaling and inositol metabolism. Analysis of the high-resolution structures from endogenous protein complexes in a close to cellular milieu (38), was enabled by the advances in cryo-EM and is an exciting field that allows for the investigation of their conformational heterogeneity (39). Our results present a unique way to probe continuous structural changes of native proteins, based on a minimal purification approach, within the context of their physiological state. We successfully identified and structurally characterized MIPS within native cell extracts, probing its activity, and thus confirming its native state. Despite the heterogenous nature of cellular extracts, we derived a high-resolution reconstruction, showcasing the power of our approach combined with modern image analysis algorithms (40). A critical insight derived from our reconstructions is the observation of the α-helical domain guarding the active site and its connection to the reaction cycle. We connect previous studies that characterized intermediate states in MIPS catalysis (14, 15, 30) with the structure of the endogenous MIPS, that has been able to clearly visualize this domain’s functions, capturing intermediate conformations in the progress.

Furthermore, we report a fully populated active site, deriving a native model for substrate binding that is in line with the previously published model concerning the prokaryotic MIPS (15).

However, there are still open questions regarding the role of an ion in stabilizing catalytic intermediates. The experimental data (*SI Appendix*, Fig. S12*A*) combined with the electrostatic analysis (Fig. 2*B*) point to the existence of a stabilizing metal ion in NAD proximity, as also seen in multiple orthologues (14, 15, 17). The identity of this ion, as well as its valence state, cannot be directly deduced. First, regarding its identity: Similar densities for prokaryotic and eukaryotic structures are interpreted by either monovalent or divalent cations (*SI Appendix*, Fig. S12*B*). Based on the active site location this ion is involved in NAD stabilization (*SI Appendix*, Fig. S12*C*) or might coordinate catalysis, as seen in other enzymatic complexes (41). The identity of this ion must be governed by its in vivo concentration; this means that Zn^+2^, although more energetically favorable (42), might not be more prevalent than the abundant Mg^2+^ (43). Our maps do not support the presence of a second ion or the widely described activator NH_4_^+^ (31). We can therefore neither rule out nor confirm a type I (44) or II aldolase mechanism for the final catalytic mechanism (45). Further structural data from endogenous MIPS across organisms are necessary to resolve this issue.

**Fig. 4.**
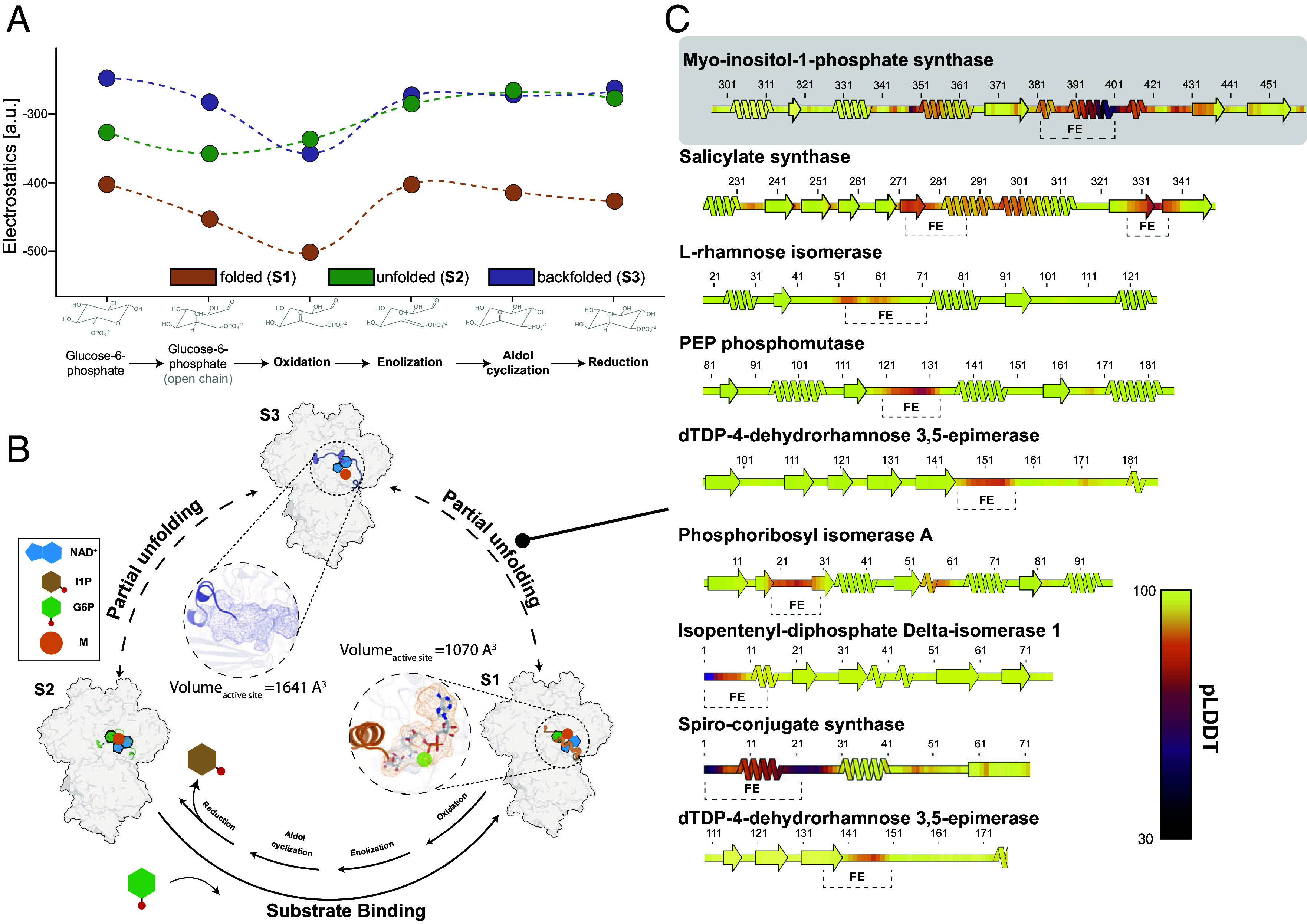
MIPS catalysis and structural conservation across isomerases. (*A*) Electrostatic interactions within the MIPS active site are the main drivers for acyclic substrate binding and stabilization. The X axis shows the reaction coordinate while the Y axis scales with electrostatics. Circles within the plot correspond to the average electrostatics of the top 4 HADDOCK models after refinement (*Materials and Methods* and *SI Appendix,* Fig. S20 for the underlying boxplots); the dotted line represents a visual component to join the energy calculations across states. Different conformations (S1 to S3) are colored with orange (S1), green (S2), and purple (S3). Other energetic contributions that remain unchanged across the reaction coordinate are shown in *SI Appendix,* Fig. S20. (*B*) The conformational selection mechanism for MIPS catalysis involving α-helix folding/unfolding transitions at the active site is displayed. Isomerization is initiated by the binding of G6P to the active site, leading to α-helix folding. NAD^+^ is tightly bound during catalysis. The reaction is catalyzed by the assembly of Lys 391, 395, and 508, converting G6P to IMP, utilizing NAD^+^. Substrate release is then initiated by α-helix unfolding, resulting in an increase in solvent accessibility as well as in screening the electrostatic interactions (both by increasing the dielectric constant and the distance between the Lys residues and the active site). A third conformation was observed, characterized by a backfolded α-helical element and an increased volume of the active site by approx. 50% in Å^3^. This state might represent the intermediate step in the observed structural transition or an autoinhibited state, driven by protein interaction or possibly posttranslational modifications. (*C*) To evaluate the conservation of active site capping via disorder-to-order transition, we analyzed all PDB-deposited isomerases, identifying enzymes where folding events correlate with active site function. Among 344 entries, corresponding to entries with more than 3 deposited structures, we identified 30 folding events linked to enzyme function. An excerpt of these transition events is shown below as a secondary structure plot. It was observed that folding events (FE) are directly linked to a slight local decrease in AlphaFold pLDDT.

Based on these findings we applied 3DVA to probe the conformational space of native MIPS to bridge the gap between structural conformation and enzymatic function (36). We succeeded to derive selected conformations and visualize the continuous disorder-to-order transition in the helical domain spanning residues 385 to 400, identifying an intermediate step characterized be the backfolding of this domain. The folding event taking place at the catalytic center is fundamental to MIPS functionality, serving as a regulatory gate, where the accessibility of the active site is governed by the population of local conformational states of the helical domain (*SI Appendix*, Fig. S17). One of the major observations is the existence of an acyclic reaction intermediate present in both states 1 and 2 but not 3 (*SI Appendix*, Fig. S17). All previous structures of bound intermediates (14, 15, 22) as well as potent inhibitors (46, 47) have been acyclic in nature. Therefore our reconstruction of the endogenous fungal MIPS, validates the claim by Frost et al. (46), proving that the unfolded state binds acyclic G6P for catalysis; which acyclic G6P is exactly in our structure is not possible to resolve; however, electrostatic energy calculations support that the first acyclic intermediate is the most stable to be found in a bound form (Fig. 4*A*).

Based on these findings, we propose the following model (Fig. 4 *A*–*C*): The catalytic cycle is initiated by the binding of acyclic G6P to the NAD^+^ preloaded active site. The reaction cycle is subsequently carried out by the assembly of Lys 391, 395, and 508, converting G6P to IMP with the help of NAD^+^. Substrate release is then initiated by α-helix unfolding, leading to an increase in reactive site volume, exposing the active site to the bulk solvent, and screening the electrostatic interactions in the process. We additionally observed a third conformation that might represent the intermediate step in the order-to-disorder transition or display an autoinhibited state, driven by protein interaction or possibly posttranslational modification (PTM). We therefore analyzed orthologue proteins for possible PTMs (*SI Appendix*, Fig. S18) and mutations. The majority of the characterized modifications introduce phosphoserine or phosphothreonine residues. Among three conserved PTM sites two residues (Ser 318, 396) were identified as inhibitory sites for enzyme function through mutational analysis (23). This is striking, considering that Ser 396 is part of the element organizing the states 1 to 3. Additionally, alanine scanning of the archaeal ortholog showed weaker activity (15) The residues reported are exactly the same in *T. thermophila*, therefore, highly conserved. One of those (Lys 278; Lys 394 in *T. thermophila*) showed local destabilization of the α-helical element, which is expected to reduce capped reactions (state S1), and therefore, catalytic activity. In addition, localized electrostatics are particularly critical for the reaction progress (Fig. 4*A*). Lys 367 (Lys 508 in *T. thermophila*) stabilizes the phosphate of the glucose-6-phosphate; again, disturbing this charge may not orient well the substrate and/or its intermediates for the reaction but its implications in selection of α-helical conformations is currently unknown. Finally, Asn 255 (Asn 372 in *T. thermophila*) may affect the activity, but its distal site in both organisms could implicate a long-range allosteric effect (*e.g.*, β-sheet interface destabilization).

Apart from the conformational cycling, we observed that the binding of NAD and G6P occurs irrespective of the conformational state clearly pointing to a mechanism beyond the reported induced fit (22). This is because binding site formation is not predominantly influenced by substrate binding, contrary to the assumptions of an induced fit model. Substrate binding acts as a stabilizing factor for the conformational intermediate, as evidenced by the electrostatic forces within the endogenous active site (Fig. 2*B*) discussed in this study. Our experimental data as well as the structural consensus point in the direction of a conformational selection mechanism (48). This proposed mechanism (Fig. 4*B*) derived from the analysis of eukaryotic, endogenous MIPS can serve as the basis for enzyme regulation and function of a plethora of analogous reactions. One such example of a direct MIPS homolog is Ari2, which governs aristeromycin biosynthesis, that shares a similar catalytic mechanism and shows an unfolded helical domain, prior to substrate binding (49). To probe possible universality for these observed conformational transitions, we compared all structurally characterized isomerases with more than three deposited structures; Such data would possibly account for conformational changes at the active site that would explain either an induced fit or/and a conformational selection model. We identified a subset of 344 isomerases, with more than 3 deposited structures (*SI Appendix*, Table S5) where approx. 10 % displayed clear indications of secondary structure change. While AlphaFold 2 predicts the conditionally folded elements (50), we observed a subtle decrease in pLDDT in the range of the folding events (Fig. 4*C*). Such folding events go beyond isomerases, as other multimeric enzymes have exhibited disorder-to-order transitions to expose or occlude their active sites, *e.g.*, the transacetylase binding site within the 60-meric core of the native and endogenous pyruvate dehydrogenase complex metabolon (51, 52). Although only analyzed for the MIPS and the isomerase class, this local and subtle decrease in pLDDT at active sites might hint conformational changes or variation; possibly, if present at distal sites, could potentially uncover allosteric events.

In conclusion, this study has considerably advanced our understanding of MIPS’s function by visualizing a conformational selection mechanism of the native enzyme at its ground state. With the integration of advanced structural biology techniques, including AI-driven structure prediction and density interpretation, bioinformatics tools, and biochemistry, we gained a more nuanced understanding of MIPS conformational heterogeneity and derived implications for its general enzyme class. It is of high importance to see these implications from the point of future therapeutic applications. MIPS is the gateway at the entry of inositol biosynthesis, and its dysregulation or malfunction has severe consequences in cellular processes (53). The provided insight into MIPS conformational shifts, its quantitative transitioning as well as its higher-order assembly, could be utilized for targeted interventions to modulate structure-based function. Based on our analysis, we expect that such active-site capping mechanisms that align with the conformational selection model for protein function underlie a plethora of enzymatic functions. We assume this because these conformational variations in the active site can regulate ground-state populations, screen localized electrostatics, and also allow solvent passage—all integral for various catalytic mechanisms at the molecular level (54–60).

## Materials and Methods

### Cultivation, Harvest, and Iysis of *T. Thermophila*.

The protocol described in ref. 51 was applied with minor adaptations (*SI Appendix*, Fig. S2). In detail, *T. thermophila* (*T. thermophila*) was grown at 52 °C and 10% CO_2_ saturation. For cultivation, Complete Culture Media (CCM), containing 3 g/l sucrose, 0.5 g/l NaCl, 0.65 g/l K2HPO4 x 3 H20, 0.5 g/l MgSO4 × 7 H20, 0.01 g/l Fe(III)sulfate-hydrate, 5 g/l tryptone, 1 g/l peptone, 1 g/l yeast cell extract, 15 g/l dextrin, and 20 g/l agar for solid culture media, were adjusted to pH 7 using NaOH. For the preculture, cultures grown for 3 d on solid culture media were used to inoculate prewarmed CCM (40 °C). For this, 200 ml of CCM was supplemented with 6 pieces of mycelium (1 × 1 cm) and incubated for 20 h at 100 rpm. Then, 800 ml of the main culture was supplemented with 15 ml preculture and incubated for 16 h, until the culture formed evenly spread mycelium globules of approx. 5 to 10 mm in diameter. The cells were then strained using a metal sieve (180 µm pore size) and washed three times with phosphate buffer saline (PBS) (pH 7.4, 4 °C). After every washing step, cells were pelleted at 3,000 *g* using a precooled centrifuge. The cell pellet was frozen with liquid nitrogen in a prechilled mortar, then ground and stored at −80 °C until used. Stored mycelium was subsequently lysed in lysis buffer, consisting of 100 mM HEPES pH 7.4, 95 mM NaCl, 5 mM KCl, 1 mM MgCl_2_, 0.5 mM EDTA, 5% glycerol, 1 mM DTT, 10 μg mL^–1^ DNAse, Pefabloc 2.5 mM, 40 μM E-64, 130 μM Bestatin, 0.5 μM Aprotinin, 1 μM Leupeptin, and 60 μM Pepstatin A. For this step, 2 g of freeze-ground mycelium was deposited on top of 10 g of beads that were preincubated in 5 ml lysis buffer and then homogenized. The sample was lysed in 3 cycles of 20 s with a Fastprep cell homogenizer at 4 °C (6.5 mps shaking speed). Between every cycle, the sample was cooled down on ice for 3 min to prevent sample degradation. The lysate was centrifuged for 5 min at 4 °C and 4,000 *g* to remove large cell debris, followed by an ultracentrifugation step for 1 h at 4 °C and 100,000 *g*. After the ultracentrifugation, the top lipid layer was removed, and the supernatant was filtered through a 0.22 µm filter.

### Size Exclusion Chromatography of Cell Extracts.

Before size exclusion chromatography (SEC), the lysate was concentrated to 30 g/l using spin filtration with a pre-equilibrated 100 kDa cutoff Amicon®-cellulose filter. Protein concentration was determined using a Bradford assay via a bovine serum albumin standard curve. Chromatography steps were carried out using the ÄKTA pure 25 M FPLC system, with a running buffer containing 100 mM HEPES pH 7.4, 95 mM NaCl, 5 mM KCl, 1 mM MgCl_2_, and 5 % glycerol. Then, 500 μl was loaded onto a Biosep SEC-S4000 column via an external loop and separated with a flow rate of 0.15 ml/min in fractions of 250 μl.

### MIPS Activity Assay.

The protocol for MIPS activity measurements has been adapted from (61). Measurements were carried out as biological triplicates with technical duplicates. The reaction buffer consisted of 100 mM Tris-acetate (pH 8), 5 mM G6P, 0.8 mM NAD^+^, 2 mM DTT, and 14 mM ammonium acetate. The assay was initiated by the addition of 10 μl (approx. 10 μg) of the respective SEC fraction, to 140 µl reaction buffer. The reaction mixture was then incubated at 37 °C for 1 h, to catalyze the conversion of G6P. Reactions were terminated with 50 µl of 20% TCA, incubating the sample for 10 min at 4 °C. To remove protein precipitate, a centrifugation step was carried out at 17.000 *g* for 30 min. For the release of inorganic phosphate, 200 µl NaIO_4_ was added to the reaction mixture and incubated at 37 °C for 1 h. A negative control representing the nonspecific release of phosphate was additionally created by omission of NaIO_4_. Phosphate release was stopped with 200 µl Na_2_SO_3_. Samples were then incubated with 120 µl of 6 M H_2_SO_4_ for 10 min at 25 °C. To initiate complex formation 240 µl H_2_0, 120 µl 2.5 % of ammonium molybdate-solution and 120 µl of 10% ascorbic acid the sample were added to the reaction mixture. The sample was incubated at 37 °C for 1 h. A phosphate standard containing 50 µl of the respective dilution (*SI Appendix*, Fig. S19), 10 µl 6 M H_2_SO_4_, 20 µl H_2_O, 10 µl 2.5 % ammonium molybdate-solution, and 10 µl ascorbic acid was additionally incubated at 37 °C for 1 h to determine the released phosphate. After the incubation period, 100 µl of the sample was transferred to a 96-well plate, and the absorbance at 820 nm was measured.

### Mass Spectrometric Protein Preparation.

For MS analysis, fractions from three distinct biological preparations were analyzed. Protein concentration of SEC fractions, derived from endogenous cell extracts, was determined via the Bradford assay. Ten micrograms of every fraction was adjusted to 100 µl using water. Then, 400 µl of acetone (−20 °C) was added to each sample, mixed, and incubated for 60 mins. Samples were centrifuged for 10 min at 17.000 *g* at 4 °C. The supernatant was then discarded, and the pellet was dried at room temperature. The protein pellet was subsequently dissolved in 10 μL of 25 mM ammonium bicarbonate (pH 8.5), containing 1% (m/v) RapiGest (Waters Corporation, Milford, USA), and incubated for 5 min at 100 °C. To reduce disulfide bridges, 10 µl 25 mM ammonium bicarbonate (pH 8.5), containing 50 mM DTT, was added. Incubation was carried out at 60 °C for 30 min. Then, 10 µl of 100 mM iodoacetamide in 25 mM ammonium bicarbonate solution (pH 8.5) was added for alkylation, incubating the sample for 30 min at 37 °C. Before proteolytic cleavage, RapiGest was diluted to 0.1% (m/v) with 25 mM ammonium bicarbonate (pH 8.5). Protein digestion was performed initially with chymotrypsin (Roche, Mannheim, Germany) at a 1:50 enzyme/protein ratio for 3 h at 24 °C. This was followed by tryptic digestion (Promega, Mannheim, Germany) at a 1:50 enzyme/protein ratio at 37 °C overnight. Hydrolysis of RapiGest was carried out by the addition of 20 *μ*l 5% (v/v) trifluoroacetic acid and incubation at 37 °C for 2 h. Samples were then centrifuged at 16,200 g for 30 min, the supernatant was collected, and the peptides were dried using a vacuum centrifuge.

### HPLC–MS.

Peptide samples were reconstituted in 2% (v/v) acetonitrile/0.1% (v/v) formic acid and analyzed by nano-flow reversed-phase liquid chromatography on a DionexUltiMate 3000 RSLCnano System coupled with a Q Exactive Plus Hybrid Quadrupole-Orbitrap mass spectrometer (Thermo Fisher Scientific, Waltham, USA).

HPLC was carried out using 0.1% (v/v) formic acid for mobile phase A and 80% (v/v) acetonitrile/0.1% (v/v) formic acid for mobile phase B. Peptides were first loaded onto a trap column (μ-Precolumn C18 Acclaim PepMap 100, C18, 300 *μ*m I.D.), particle size 5 *μ*m, (Thermo Fisher Scientific, Waltham) with a flow rate of 10 *μ*l/min. Separation was then performed on an analytical C18 capillary column (50 cm, HPLC column Acclaim PepMap 100, C18, 75 *μ*m I.D.), particle size 3 *μ*m, (Thermo Fisher Scientific, Waltham, USA) with a flow rate of 300 *n*l/min. A gradient of 4 to 90% (v/v) mobile phase B over 90 min was applied. Eluting peptides were analyzed under the following mass spectrometric conditions: data-dependent mode, Data-dependent mode, capillary voltage of 2.8 kV, transfer capillary temperature of 275 °C, samples were measured in positive ion mode. Survey full scan MS spectra were acquired in a mass range of 350 to 1600 m/z, with a resolution of 70,000 setting the automatic gain control (AGC) target at 3 × 10^6^. The maximum injection time was set to 80 *m*s. The 20 most intense peaks were selected for fragmentation in the HCD cell with an AGC target of 1 × 10^5^, a fixed first mass of 105 m/z, and a normalized collisional energy of 30%. The maximum injection time for MS2 spectra was 150 *m*s, and the resolution of MS2 spectra was 17,500. Ions with a charge of 1 and > 8 were excluded from fragmentation, and previously selected ions were dynamically excluded for 30 s. For internal mass calibration, the lock mass option was enabled using the lock mass m/z 445.120025 (62).

### MS Data Analysis Using MaxQuant.

MaxQuant (version 1.6.17.) (63) was used for analysis of raw MS data, using the proteome of *T. thermophila* as a reference (UniProt Proteome ID: UP000008066). Carbamidomethylation of cysteines was set as a fixed modification, while oxidation of methionine and N-term acetylation was set as a variable modification. Maximal missed cleavage sites were set to 2, minimal peptide length to 7 and maximal peptide mass to 6,000 Da. Peptide and protein FDR were defined as 0.01. Enzymes for proteolytic cleavage were set to trypsin/P and chymotrypsin. All fractions from the biological triplicate were set as independent experiments in one MaxQuant database search. Calculated LFQ intensities of triplicates were averaged to determine the abundance of eluting complexes across fractions.

### Cryo-EM Sample Preparation and Data Collection.

For vitrification, Quantifoil® type R2/1 holey carbon-coated support films on copper 200 mesh grids were used. The grids were glow discharged using a PELCO easiGlow™ at 15 *m*A and 0.4 bar for 25 s of glow time. Then, 3.5 µl of the sample, with a concentration of 0.6 g/l, was applied to each EM-grid. For vitrification, the Vitrobot Mark IV system was used. The blotting paper (ø55/20 *m*m and ash-free Grade 595 filter paper) was loaded before equilibrating the Vitrobot at 4 °C and 100% humidity. The blot force was set to 2 and the blotting time to 6 s. Then, the grids were plunge-frozen in liquid ethane, clipped to the manufacturer’s specifications, and loaded into the Thermo Fisher Scientific (TFS) Glacios cryotransmission electron microscope(cryo-TEM), equipped with a Falcon 3EC direct electron detector. For data acquisition, the movies were recorded with a dose of 28 e/Å^2^, in a defocus range of −2.5 to −1 *μ*m, and a pixel size of 0.59 Å (240.000 magnification). A total of 6,261 movies were recorded in one session.

### Image Processing of Cryo-EM Data.

Image processing was performed using the CryoSPARC software package v3.3.2 (40). An extended schematic of the image analysis pipeline is shown in *SI Appendix*, Fig. S5. In short, movies were motion corrected using patch motion correction (multi), and a patch CTF estimation (multi) was performed for all 6,261 micrographs. Corrected micrographs were then subjected to reference-free blob picking, with a defined particle diameter range of 100 to 250 Å, resulting in 2,054,291 particle picks. Picked particles were then extracted with a box size of 416 pix and subjected to reference-free 2D classification in 150 classes. All “junk” classes were discarded, and prominent particle signatures were reclassified until 3 distinct signatures, emerged. Signature I, signature II, and signature III were then used for ab initio reconstruction and homogeneous refinement without applying symmetry. Signature I was initially reconstructed at 3.1 Å, signature II at 9.4 Å, and signature 3 at 3.6 Å. Then, 50 equally spaced template projections from the derived volumes were used for template picking, applying a lowpass filter of 20 Å to both micrographs and templates, resulting in 308,392 particles for signature I, 53,588 particles for signature II, and 19,420 particles for signature III, after 3 rounds of template picking and classification. To correctly identify particle signatures, the corresponding volumes were compared to the structures of the top 25 most abundant protein hits, based on their LFQ value.

Particles belonging to signatures I, II, and III were identified as the fungal MIPS, pyruvate kinase (PK), and glutamine synthas (GS). Particles were then used for ab initio and homogenous reconstruction. The aligned particles as well as the reconstructed volume were used for reference-based motion correction. Then, 255.354 particles were used for nonuniform refinement with D2 symmetry, reaching 2.48 Å (FSC = 0.143) (*SI Appendix*, Fig. S6*A*). A local resolution estimation revealed a local resolution range of 2 to 3 Å. Additionally, 3D variability analysis (3DVA) was performed to probe the particle set (signature I) for heterogeneity. For this, particles used for homogenous refinement prior to 3D classification were symmetry expanded (D2), and a local refinement was performed. The aligned particles as well as the generated mask of the local refinement were used for 3DVA with 3 components at a filter resolution of 5 Å. The component displaying the most variance in the region surrounding the active site was then used for 3D variability display, setting the number of clusters to 5, the filter resolution to 5 Å, and the output mode to cluster. Clusters were then assigned to different conformational states based on their corresponding volumes, resulting in 23% of the particles assigned to state 1, 69% of the particles to state 2, and 8% of the particles to state 3. All particles from states 1 and 3 were then used for local refinement, resulting in a final reconstruction at 2.8 and 3.2 Å, respectively (FSC = 0.143) (*SI Appendix*, Figs. S14*A* and S15*A*). For map visualization, the software package Chimera X (64) was used.

### AI-Based Model Generation and Refinement.

AlphaFold-multimer, run in a local installation, was used for the predictions of the tetrameric assemblies of fungal (Uniprot ID: G0SDP4) and human MIPS (Uniprot ID: Q9NPH2) (65). Models were relaxed using the AMBER force-field option. AlphaFold quality statistics [predicted aligned error (PAE) and predicted local distance difference test (PLDDT)] are shown in *SI Appendix*, Fig. S8*A*. For subsequent model building, regions of low pLDDT score (<70) were cut from the model for further refinement. Truncated models generated by AlphaFold were fitted inside the EM reconstructions, using ChimeraX (64). Models were subsequently real space refined using the PHENIX software package (66), applying secondary structure as well as map symmetry restraints. The monomeric model was manually refined in COOT (67) and map NCS operators were applied, using PHENIX. Model quality was analyzed using MolProbity (68) integrated in PHENIX to iteratively improve the model. Different substrates for the binding position of glucose-6-phosphate were evaluated (*SI Appendix*, Fig. S13*B*) using the UCSF Chimera plugin MAPQ (34) to calculate Q-scores. Surface electrostatic potential was calculated directly in ChimeraX. Active site volumes were calculated using the PyMOL (Schrödinger, USA) plugin PyVOL (69), only selecting the largest volume, setting the minimum radius to 1.4 and the maximum to 3.4.

### Energetic Calculations.

The three-step energetic calculation protocol was performed on the HADDOCK2.2 web server (70). For interface energetics, MIPS structures were retrieved from the PDB, selecting prokaryotic as well as eukaryotic structures, omitting mutant structures. Additionally, the AI-predicted structures for human MIPS (*SI Appendix*, Fig. S7*B*) as well as the final reconstruction of state 2 were included for the comparison. Active site energetics were calculated based on the respective, real space refined models of the reaction intermediates (*SI Appendix*, Fig. S20). The protocol is based on the already described refinement methodology (71) and was the same as the one we previously applied for another enzymatic complex (72). Important aspects of the protocol are the utilization of the Berendsen thermostat (73) to maintain constant temperature during the simulation; the Crystallography and NMR Software (CNS) which serves as a computing engine (74); and the applied OPLS force-field (75) for integration of equations of motion and calculation of related energetic components.

### Multiple Sequence Alignments and Secondary Structure Prediction.

The following sequences for MIPS were retrieved from the Uniprot database: G0SDP4 (*T. thermophila*), P9WKI1 (*M. tuberculosis*), P11986 (*S. cerevisiae*), G5ED01 (*C. elegans*), Q9NPH2 (*H. Sapiens*), Q38862 (*A. thaliana*), Q8A7J8 (*H. thetaiotaomicron*), and O28480 (*A. fulgidis*). The sequences were aligned using MUSCLE with default settings (76). Conservation and the consensus sequence were visualized with Jalview (77). Alignments are displayed in *SI Appendix*, Fig. S21. To probe folding propensity of the helical domain between residues 385 and 405, multiple secondary structure prediction algorithms were applied to the fungal and prokaryotic sequence (*SI Appendix*, Fig. S11). pLDDT scores were retrieved via the AlphaFold Protein Structure Database (78). SecStr (79), containing the algorithms of Nagano, Garnier, Burgess, Chou and Fasman, Lim, Dufton and Hider, as well as PSIPRED (80), SPIDER 3 (81), PSSPRED4 (82), DEEPCNF (83), and NETSURFP2 (84) were used. Secondary structure plots were created using SSDraw (85).

## Supplementary Material

Appendix 01 (PDF)

Dataset S01 (XLSX)

## Data Availability

1) EM MAP (.MRC) 2) PDB FILES (.PDB) 3) PROTEOMIC DATA 4) supplement MAPS (.MRC), supplement PDB Files (.PDB), & HADDOCK energy calculations data have been deposited in 1) EMDB 2) PDB 3) PRIDE 4) SBGrid. 1) EMDB: 50149 (86) 2) PDB: 9F2K (87) 3) PRIDE: PXD048355 (88) 4) SBGrid: 1105 (89)). The 3D maps (half-maps, refined map) of state 1 are available at EMDB database under the accession code EMD-50149 (86), the molecular model of state 1 MIPS at the PDB database under the accession code PDB ID: (9F2K) (87). Maps and models of the alternative states, as well as the datasets for the energy calculations, are deposited in the SBGrid database under the accession code 1105. The mass spectrometry proteomics data have been deposited to the ProteomeXchange Consortium via the PRIDE (90) partner repository with the dataset identifier PXD048355 (88).
