# Supplementary material for: Disorder-to-order active site capping regulates the rate-limiting step of the inositol pathway
Source: Proc Natl Acad Sci U S A. 2024 Aug 15;121(34):e2400912121. doi: 10.1073/pnas.2400912121 (PMC11348189; doi:10.1073/pnas.2400912121)
Supplement: Supplementary file 1 — Appendix 01 (PDF) [file pnas.2400912121.sapp.pdf]

PNAS

Supporting Information for

Disorder-to-order active site capping regulates the rate-limiting step of the inositol pathway

Toni K. Träger[1,2], Fotis L. Kyrilis[3], Farzad Hamdi[1,2,4] Christian Tüting[1,2,4], Marie Alfes [4,5], Tommy Hofmann[4,6], Carla Schmidt[1,4,7] & Panagiotis L. Kastritis[1,2,3,4].

[1]Institute of Biochemistry and Biotechnology, Martin Luther University Halle-Wittenberg, Kurt-Mothes-Straße 3, 06120, Halle/Saale, Germany

[2]Biozentrum, Martin Luther University Halle-Wittenberg, Weinbergweg 22, 06120, Halle/Saale, Germany

[3]Institute of Chemical Biology, National Hellenic Research Foundation, Athens, 11635, Greece

[4]Interdisciplinary Research Center HALOmem, Charles Tanford Protein Center, Martin Luther University Halle-Wittenberg, Kurt-Mothes-Straße 3a, 06120, Halle/Saale, Germany

[5] Biologics Analytical R&D, AbbVie Deutschland GmbH & Co. KG, Knollstraße, 67061 Ludwigshafen, Germany

[6] IDT Biologika Dessau-Roßlau, Am Pharmapark, 06861 Dessau-Roßlau, Germany

[7]Department of Chemistry─Biochemistry, Johannes Gutenberg University Mainz, Biocenter II, Hanns-Dieter-Hüsch-Weg 17, 55128 Mainz, Germany.

*Panagiotis L. Kastritis

Email: panagiotis.kastritis@bct.uni-halle.de

This PDF file includes:

Figures S1 to S21
Tables S1 to S5
SI References

Other supporting materials for this manuscript include the following:

Dataset S1

Fig. S1. MIPS cellular environment and native purification. **(A)** Myo-inositol-phosphate synthase (MIPS) is the rate-limiting enzyme in the inositol pathway. Glucose-6-phosphate (G6P) derived from the hexokinase is converted to inositol-1-phosphate (I1P) in a 5-step reaction by MIPS (blue). After ring opening, G6P is oxidized by NAD+ to form a keto group at the C5 position. Enolization at the C5-C6 carbon bond leads to the aldol cyclization. NAD+ is regenerated by the reduction of the C5 carbon. **(B)** The thermophilic fungus *Thermochaetoides thermophila* (*Th. t.*) is utilized for the generation of cell extracts, combining the advantages of eukaryotic systems with the increased protein stability compared to mesophilic organisms (1).

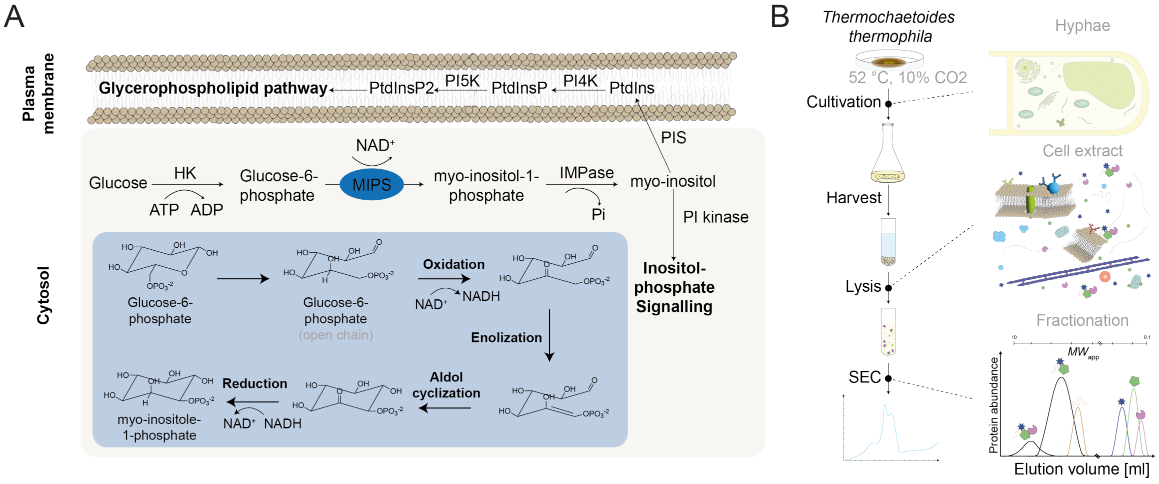

Fig. S2. Preparation of fractionated cell extracts and associated quantities. The schematic workflow for the production of cell extracts, adapted from (2),is displayed. In short, after cultivation the mycelium is harvested. Lysis is carried out by bead beading, followed by two centrifugation steps at 4000 and 100000 *g*. The lipid film is removed, the supernatant is collected and filtered through a 0.22μm filter syringe tip filter. The cell extract is then concentrated and separated by SEC.

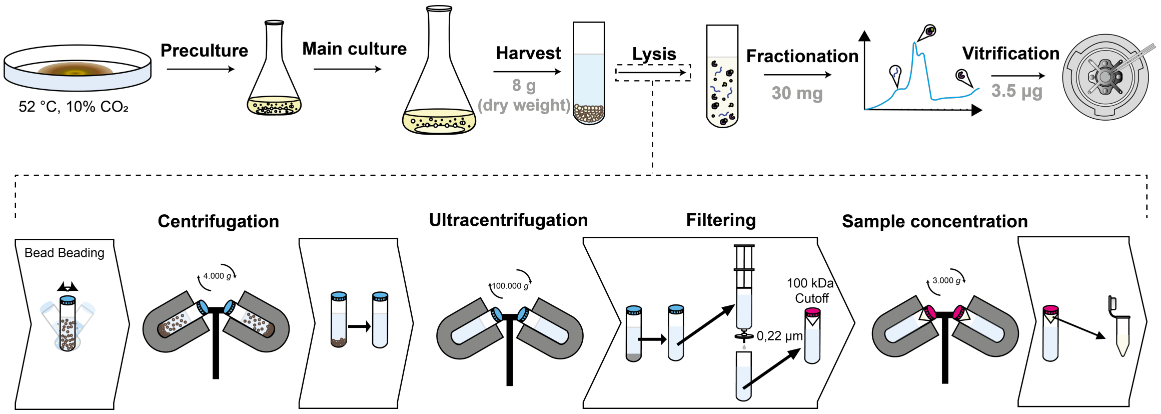

Fig. S3. Analysis of native cell extracts. (A) Crude cell extracts were fractionated using size exclusion chromatography (SEC). Retention times of the standard proteins thyroglobulin (669 kDa), ferritin (440 kDa), and aldolase (158 kDa) are shown. Screened fractions are marked in blue. (B) Activity of endogenous MIPS per fraction. Individual fractions are plotted against normalized reaction rate. To identify retention times of MIPS and characterize coeluting proteins label free MS was carried out on a biological triplicate of SEC fractions. MIPS, glutamine synthase (GS), and pyruvate kinase (PK) were identified as the structurally most abundant signatures based on their relative abundance intensities (LFQ score, top). (C) A representative micrograph of the fraction where MIPS was abundant and exhibited the highest relative activity is shown (fraction 35), including several other structural signatures (arrows). (D) 2D class averages of GS (green), PK (blue), MIPS (black) as well as 2 more unidentified proteins (orange and pink).

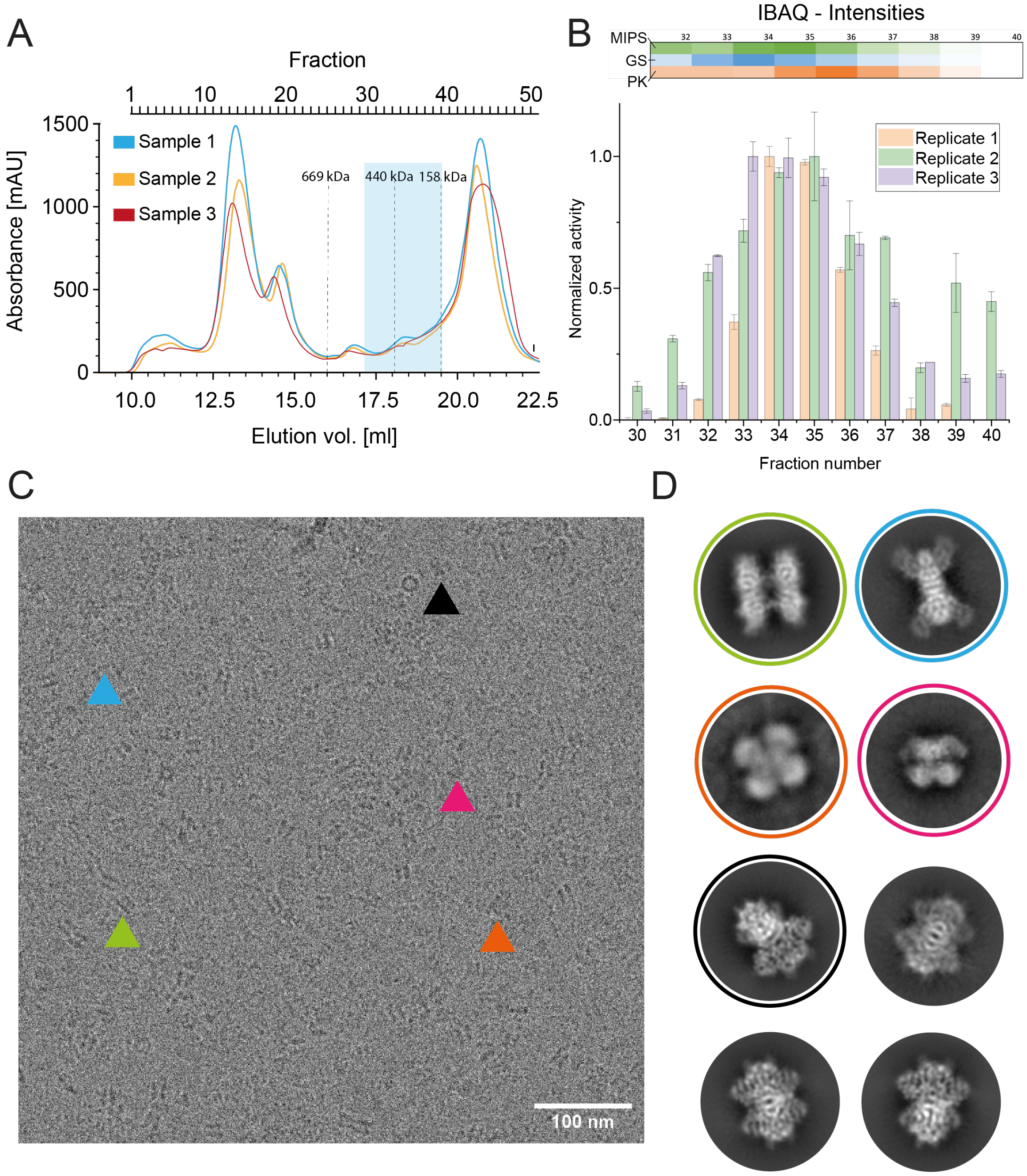

Fig. S4. Composition of native cell extracts. Label-free quantitative mass spectrometry was utilized for an alternative readout of the sample heterogeneity for the analyzed fraction 35. (A) A protein network analysis based on STRING interaction data (3) was performed, revealing a complex network of higher-order interaction present in the analyzed fraction. (B) The 20 most abundant proteins based on their respective itensities were plotted, showing MIPS to be one of the major constituents of fraction 35.

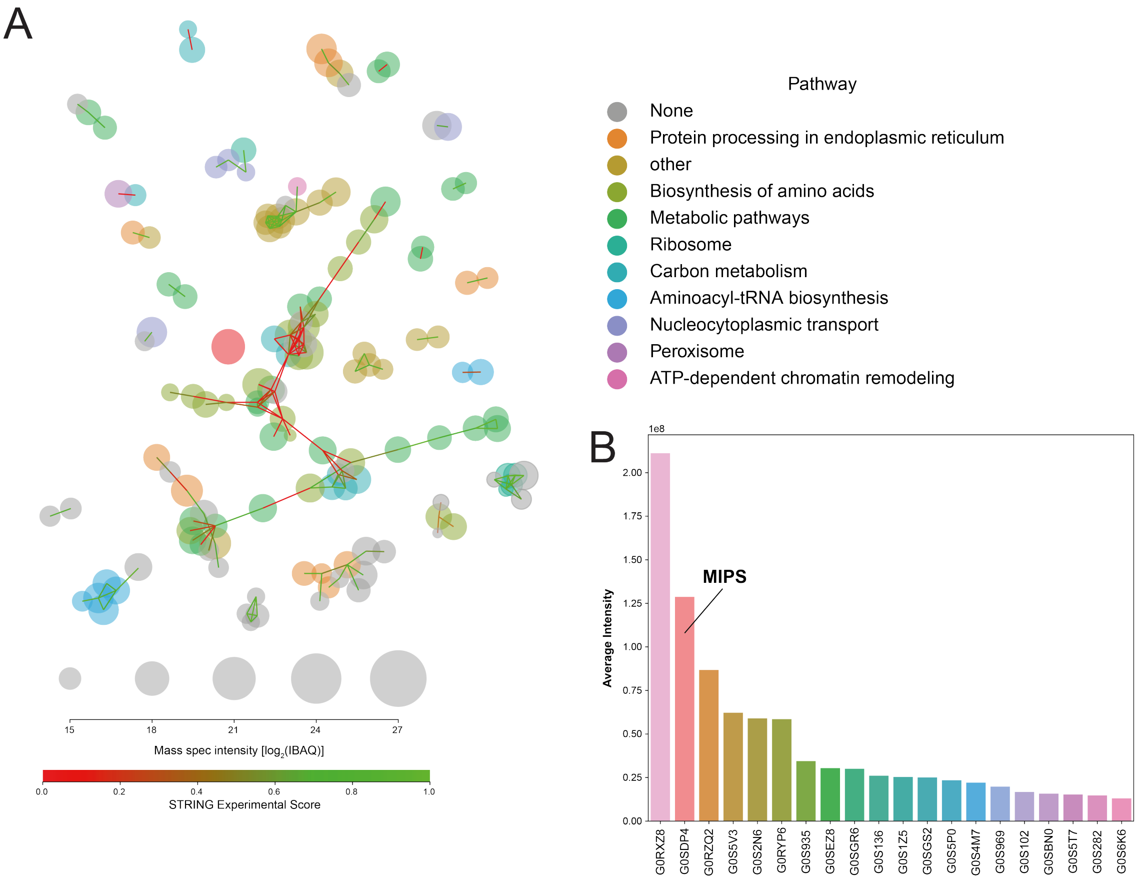

Fig. S5. Image analysis workflow. Schematic representation of the image analysis pipeline.

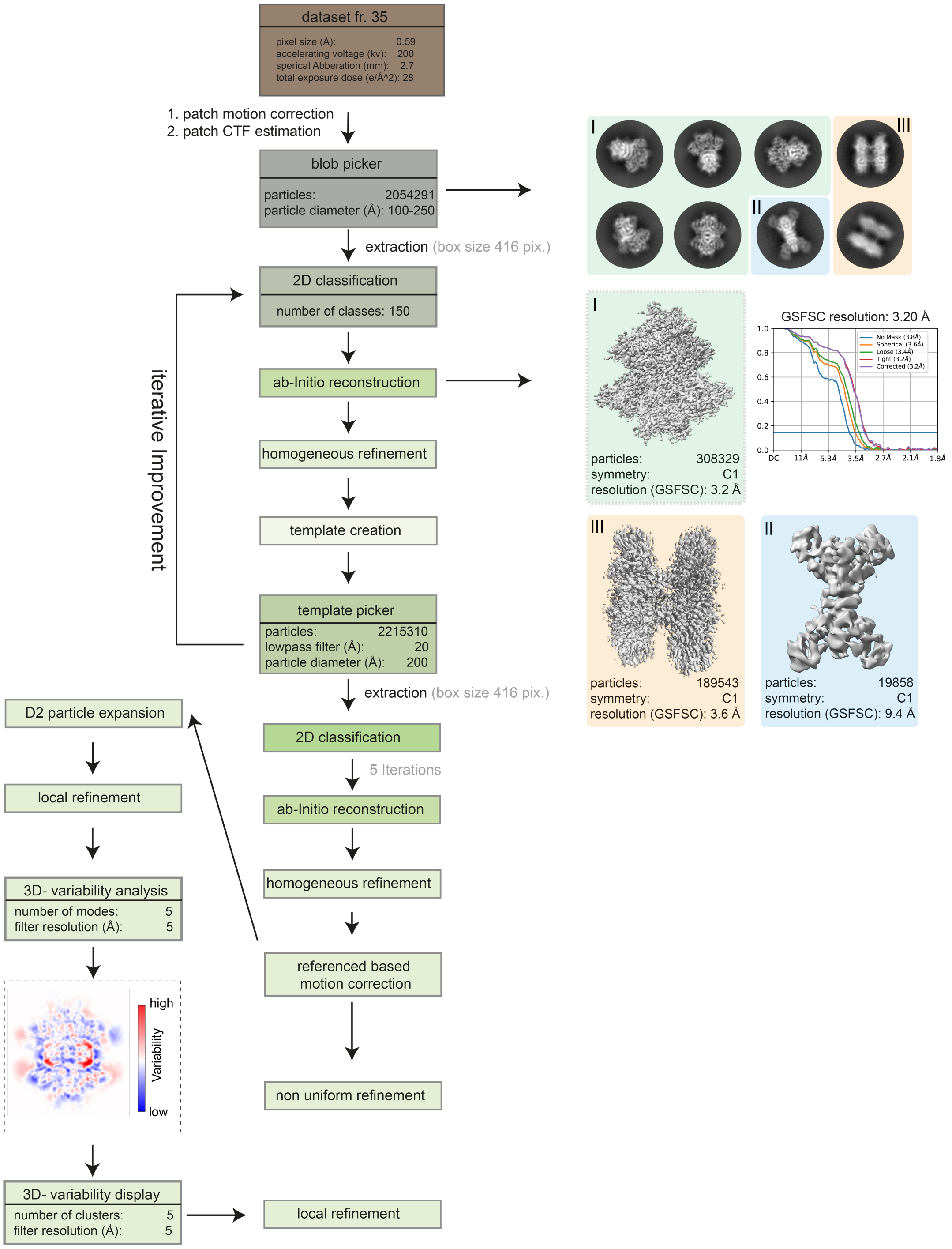

Fig. S6. Quality statistics for the reconstruction of endogenous MIPS. (A) The final refinement (D2 symmetry) included 255,354 particles. The gold standard FSC (0.143) was calculated to be 2.48 Å. (B) The mask used for the determination of the average resolution at FSC 0.143 is displayed. (C-D) The view coverage and the directional FSC (3D FSC) (4) are shown. (E) The local resolution estimation of the reconstructed volume shows a distribution between 2 and 3 Å, with rare occurrences up to 3 Å (contour level 0.2).

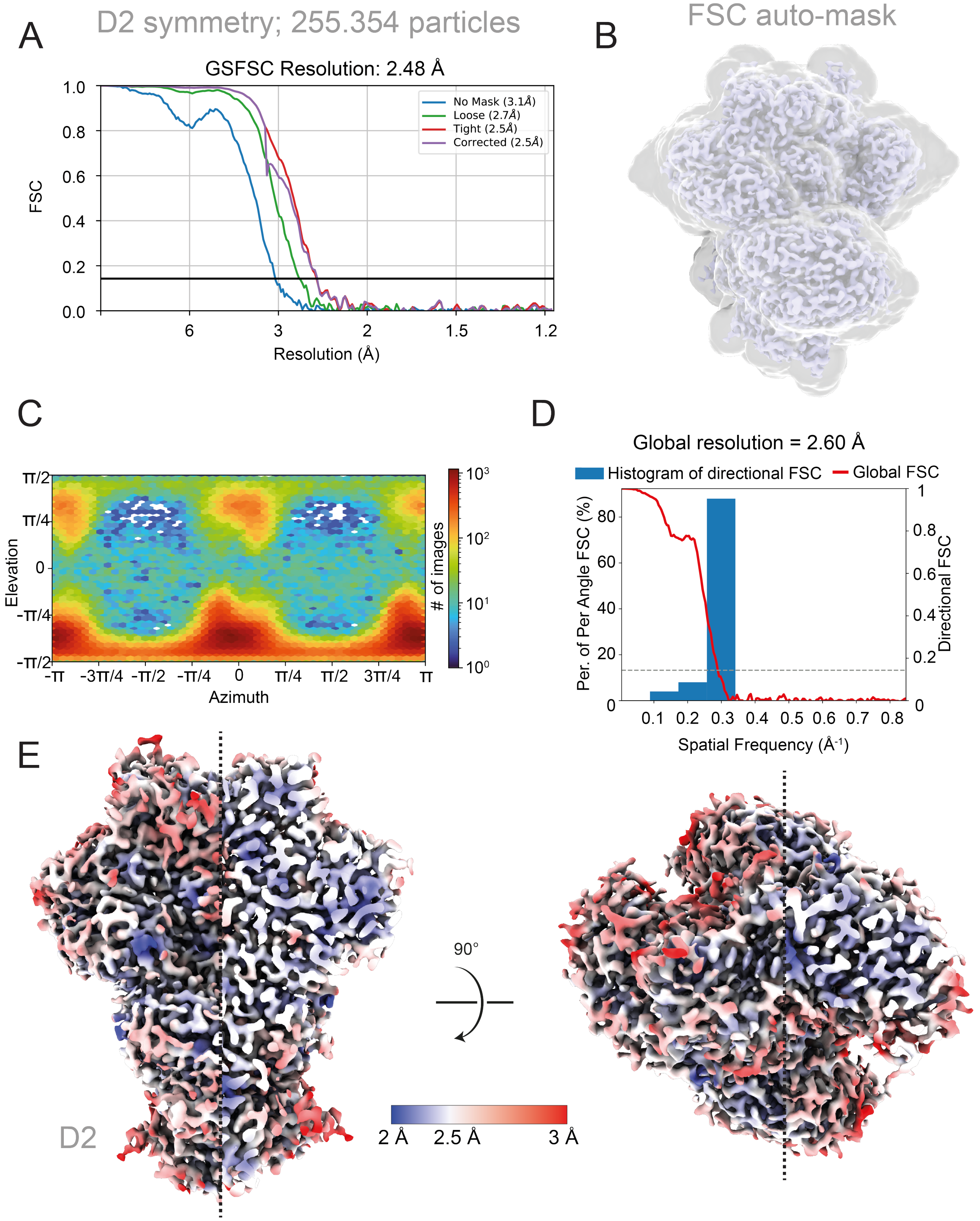

Fig. S7. AlphaFold model analysis. Models of MIPS from *Thermochaetoides thermophila* (A) (Uniprot ID: G0SDP4) and *Homo sapiens* (B) (Uniprot ID: Q9NPH2) were generated using AlphaFold 2 (5). Per-residue estimate of its confidence (pLDDT) and Predicted Aligned Error plot, for the confidence in the domain packing and large-scale topology, as well as the template sequence coverage for the best scoring model are displayed.

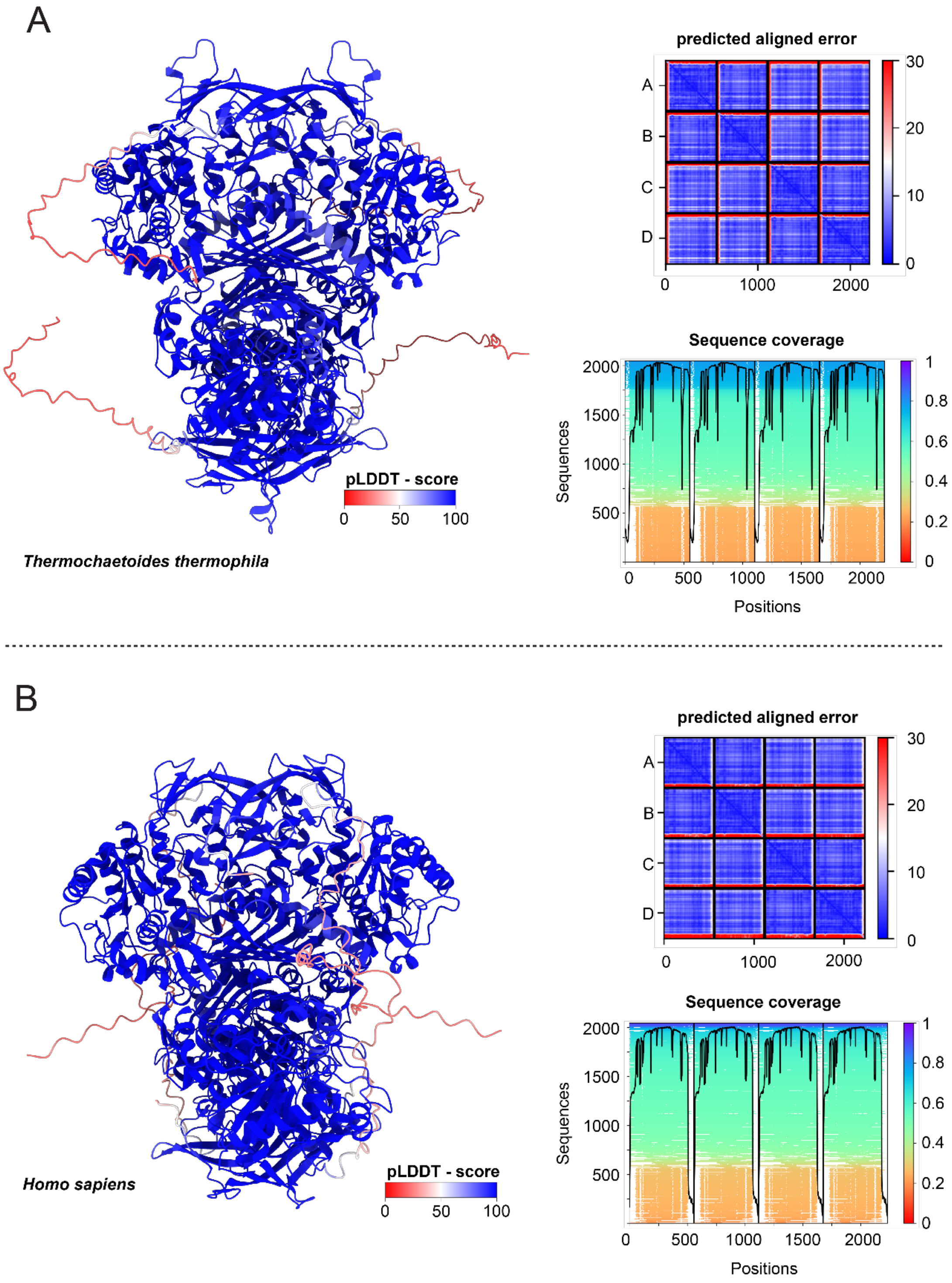

Fig. S8. MIPS is partially disordered. (A) To probe the evolutionary conservation of the N-terminal disordered region in fungal MIPS, orthologs from different organisms were compared using their AlphaFold 2 predictions (5). Although not a direct identifier for disordered regions, the pLDDT score correlates with low complexity regions, showing a clear complexity shift from N-terminus to C-terminus, while being completely absent in plants. (B) Disordered regions often serve as mediating regions for protein-protein interactions. To elucidate possible cellular interactors, the interactome of fungal MIPS was analyzed using the STRING database (3), revealing a plethora of possible targets, few of them also recovered in the fractionated extract co-eluting with MIPS.

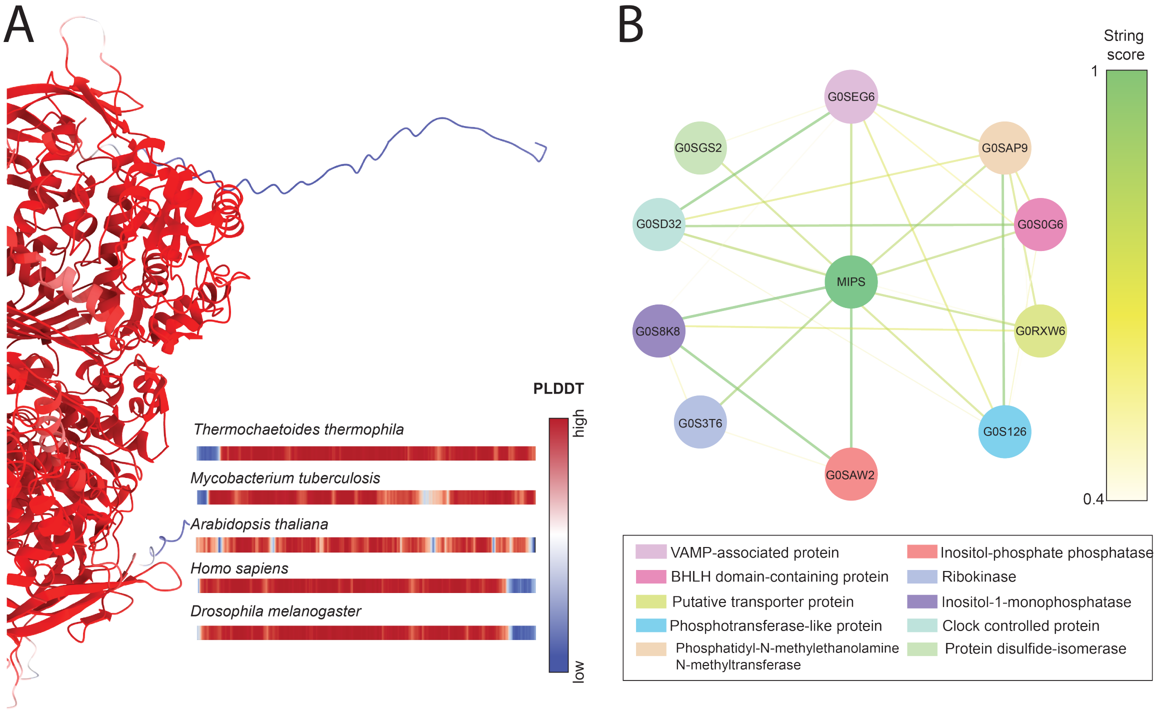

Fig. S9. Structural organization across MIPS orthologues. (A) To compare the thermophilic MIPS against its mesophilic counterpart, MIPS from *S. cer.* (PDB ID: 1JKI) (6) was overlaid on the final reconstruction. (B) To visualize the contrasting conformations between both models, all-atom root-mean-square deviation (RMSD, per residue, in Å) was computed. This analysis highlighted several areas with shifts that exceeded 8 Å (Ångstroms). Loop regions in multiple regions are shortened and compacted in the thermophilic structure (insert; contour level 0.075).

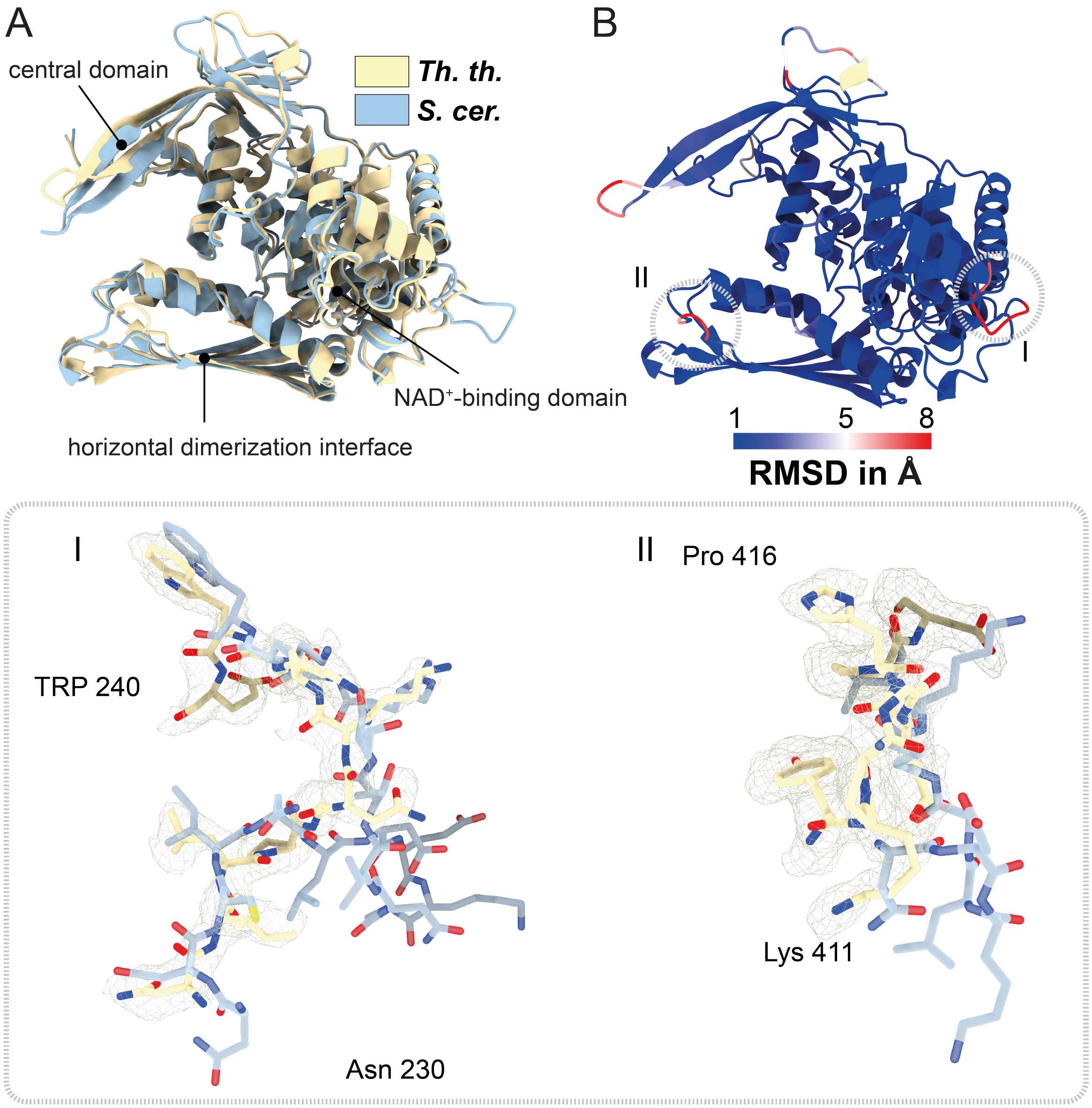

Fig. S10. Energetics of MIPS across species. (A) Horizontal and vertical interfaces of the tetrameric assembly are stabilized by a plethora of electrostatic and hydrophobic interactions. Notable interactions of the interfaces are displayed in sticks and utilizing the electrostatic surface potential map representation (contour level 0.2). (B) To identify the driving forces behind MIPS assembly, horizontal (blue) and vertical (orange) dimeric interfaces were energetically characterized using molecular refinement procedures. Red dot is MIPS energetics, other dots represent all other crystallographically determined MIPS structures (see *SI Appendix,* Tab. 2 and 3 for details).

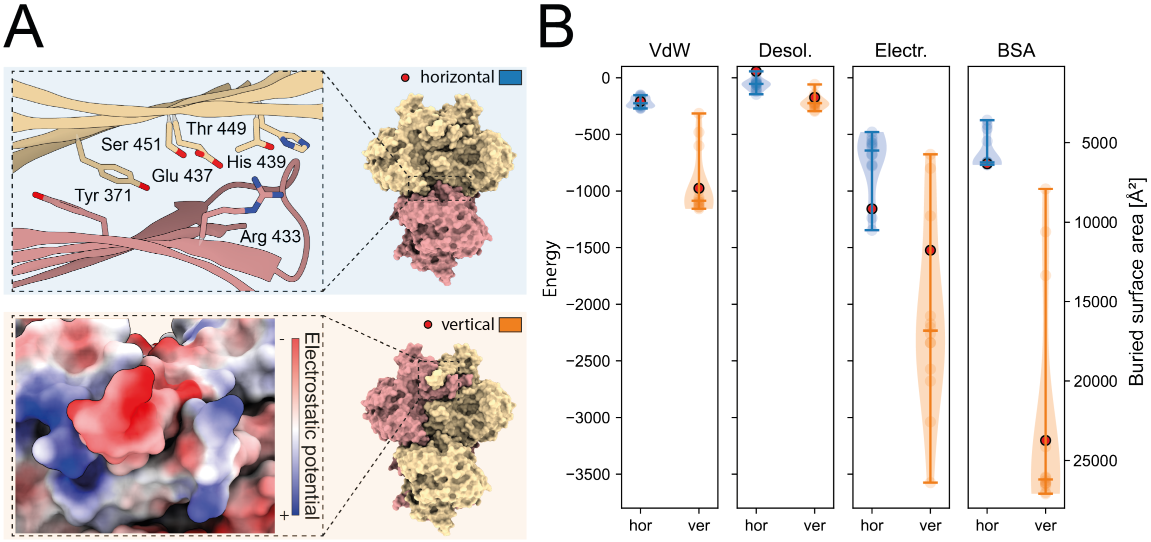

Fig. S11. Secondary structure prediction of MIPS helical domain. To identify the propensity of helical domain formation at the entry of the active site, sequences representing the eukaryotic MIPS from *T. thermophilia* (residues 385 to 405) and the prokaryotic *A. fulgidis* were compared. Consensus between all secondary structure prediction methods is shown. pLDDT scores were taken from AlphafoldDB (7).

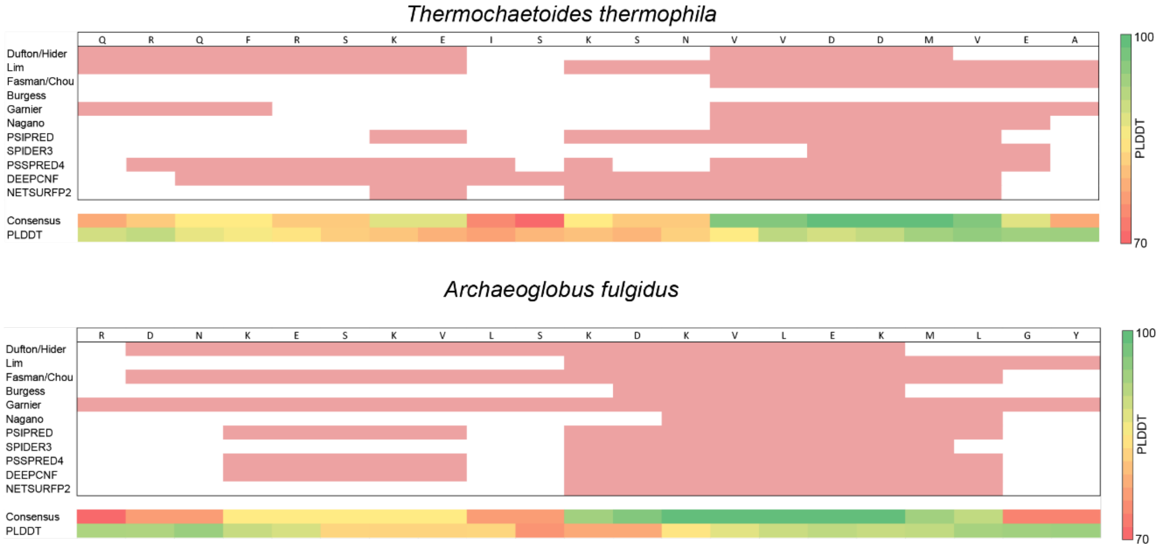

Fig. S12. Homology-based modeling of ionic interactions. (A) Apart from its two cofactors, the active site of MIPS revealed an additional signature with scaling properties that were not in line with water residues (respective contour levels are stated). (B) To identify the most likely candidate for this, orthologue structures were aligned, pointing in the direction of a metal ion that is positioned between the headgroup and the diphosphate group of NAD. (C) Ion interaction network, with its respective distances, is displayed.

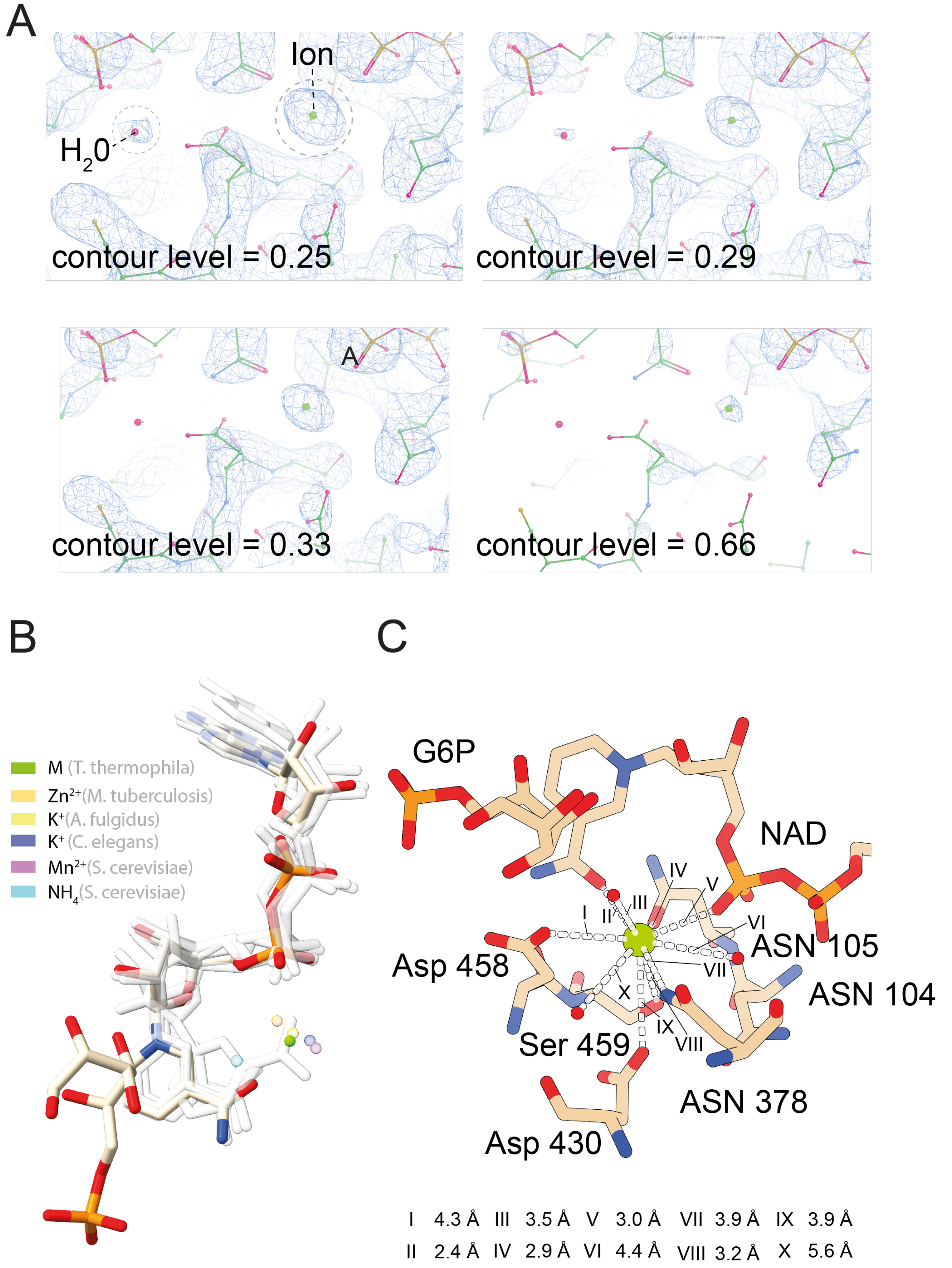

Fig. S13. Analysis of active site substrates. (A) Close analysis of the active site of the published eukaryotic MIPS structures revealed a conformational stabilization mechanism for NAD, related to the binding of conserved Ion. The distance between the nicotinamide and phosphate groups was measured. (B) To evaluate the possible substrates for the active site of the fungal MIPS, all reaction intermediates were real-space-refined inside the reconstruction. The final structure was then validated via Q-scoring (8), revealing higher cross-correlations with the EM density for the accommodation of the acyclic intermediates (contour level 0.075).

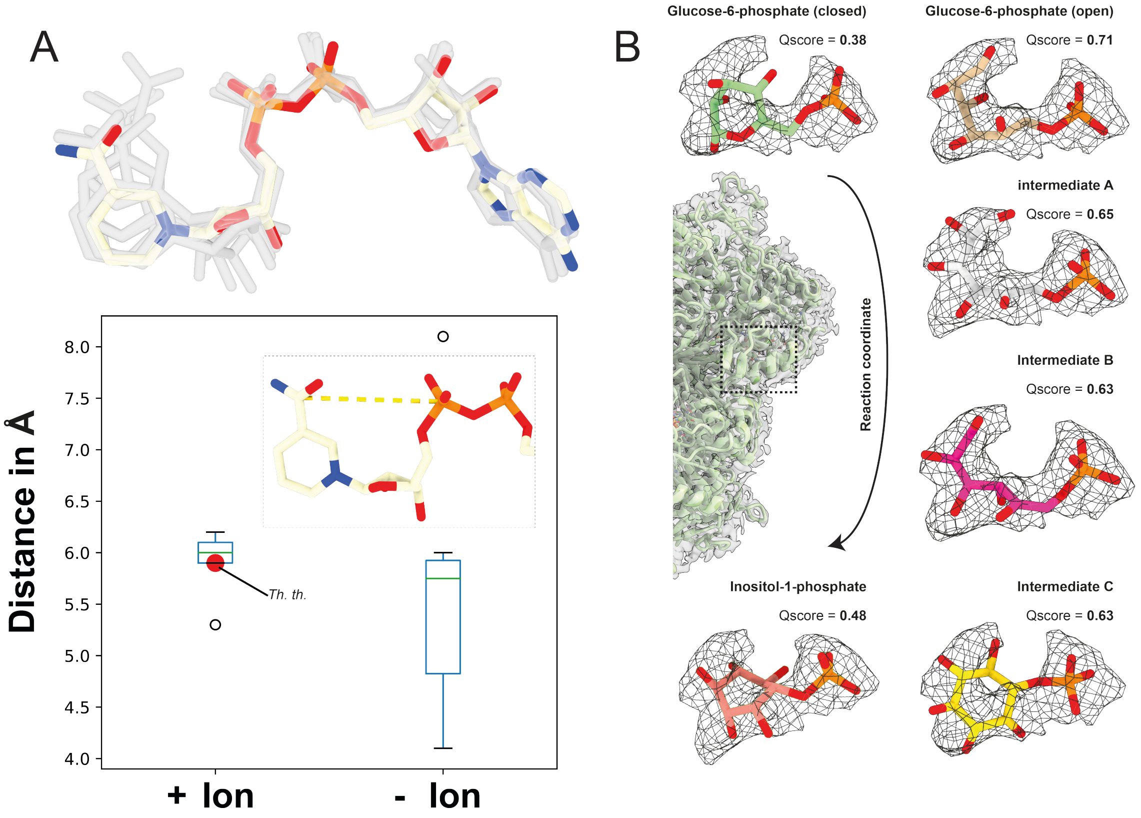

Fig. S14. Quality statistics for the reconstruction of endogenous MIPS in the unfolded state. (A) The final refinement included 58,544 particles (D2 symmetry). FSC (0.143) was calculated to be 2.8 Å. (B) The mask used for the determination of the average resolution at FSC 0.143 is displayed. (C-D) The view coverage and the directional (3D FSC) (4) are shown. (E) The local resolution estimation of the reconstructed volume shows a distribution between 2 and 6 Å (contour level 0.2).

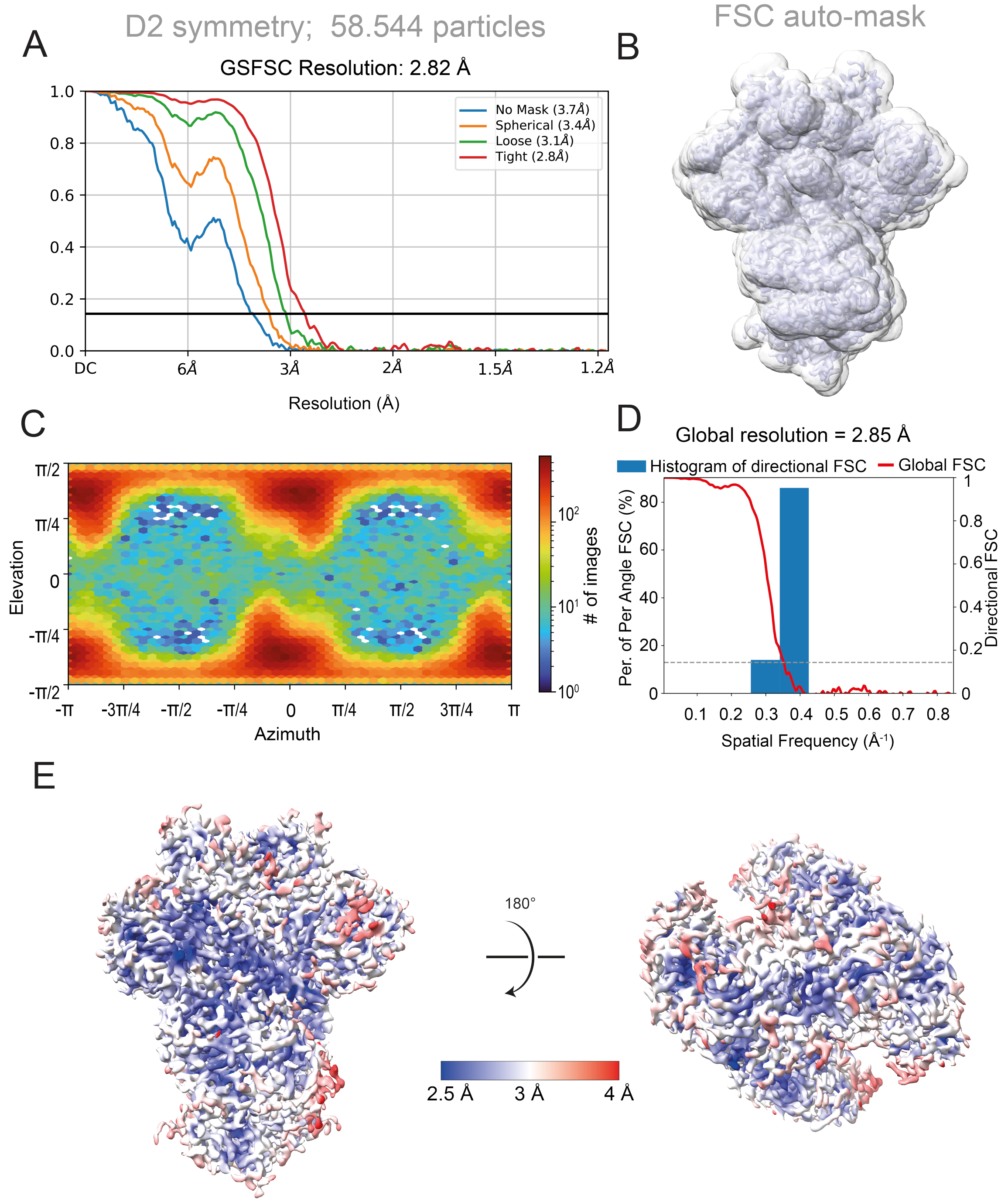

Fig. S15. Quality statistics for the reconstruction of endogenous MIPS in the backfolded state. (A) The final refinement included 19,408 particles (D2 symmetry). The gold standard FSC (0.143) was calculated to be 3.2 Å. (B) The mask used for the determination of the average resolution at FSC 0.143 is displayed. (C-D) The view coverage and the directional (3D FSC) (4) are shown. (E) The local resolution estimation of the reconstructed volume shows a distribution between 2 and 6 Å (contour level 0.2).

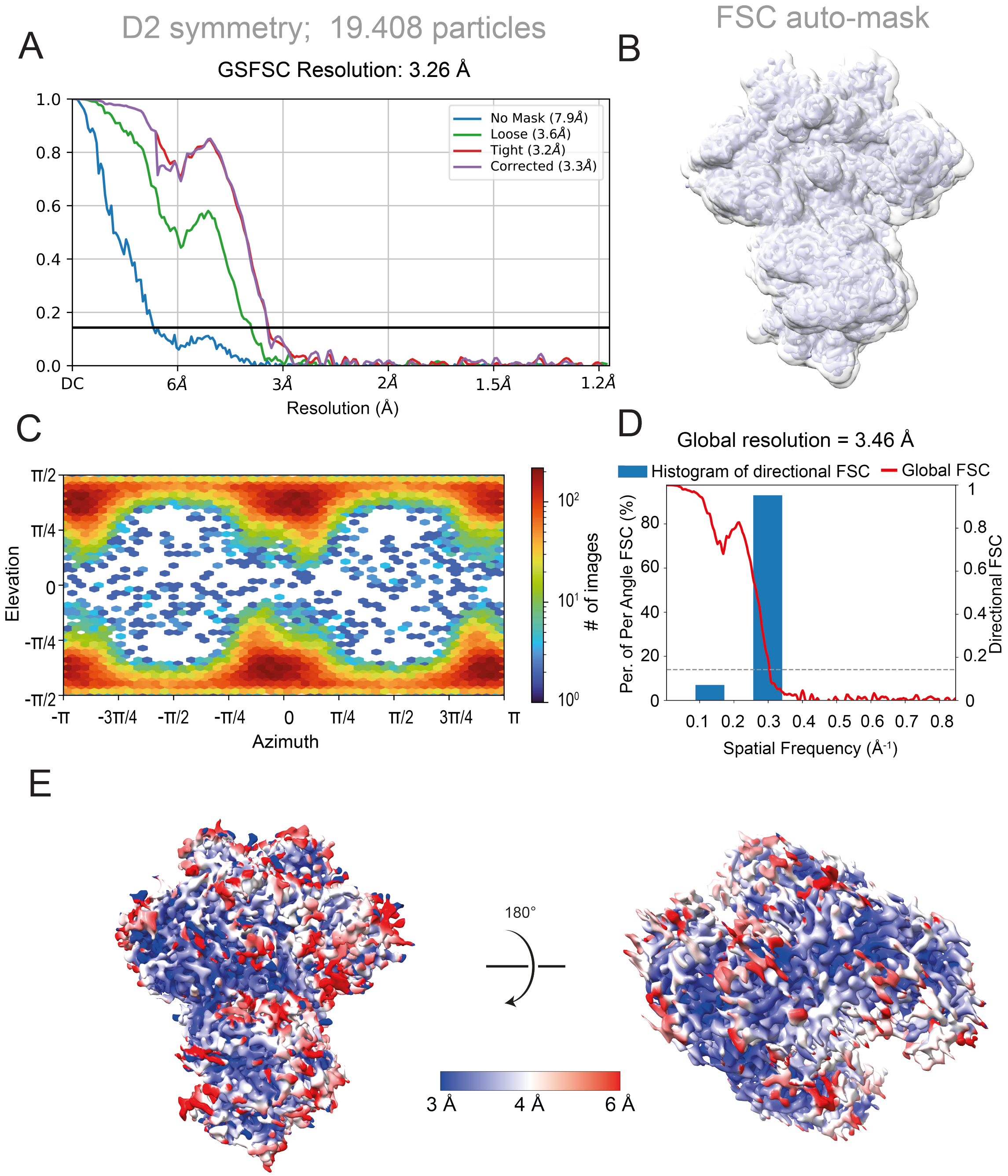

Fig. S16. Comparison of MIPS states. (A) 3DVA (9) revealed an alternative state for MIPS (state 3), characterized by the absence of the α-helical domain at the entry of the active site, visible in state 2 (contour level 0.08). A backfolded domain with a displacement greater than 5 Å, compared to the α-helical domain of state 2, is visible (yellow cross). (B) Model of state 2 was fitted into the reconstruction of state 3, clearly displaying the absence of a α-helical domain at the active site (contour level 0.2). The model derived from the medium resolution reconstruction of state 3 was compared to the model for state 2, revealing regions of conformational change. (C) To visualize the contrasting conformations between both states, root-mean-square deviation (RMSD) per residue was computed.

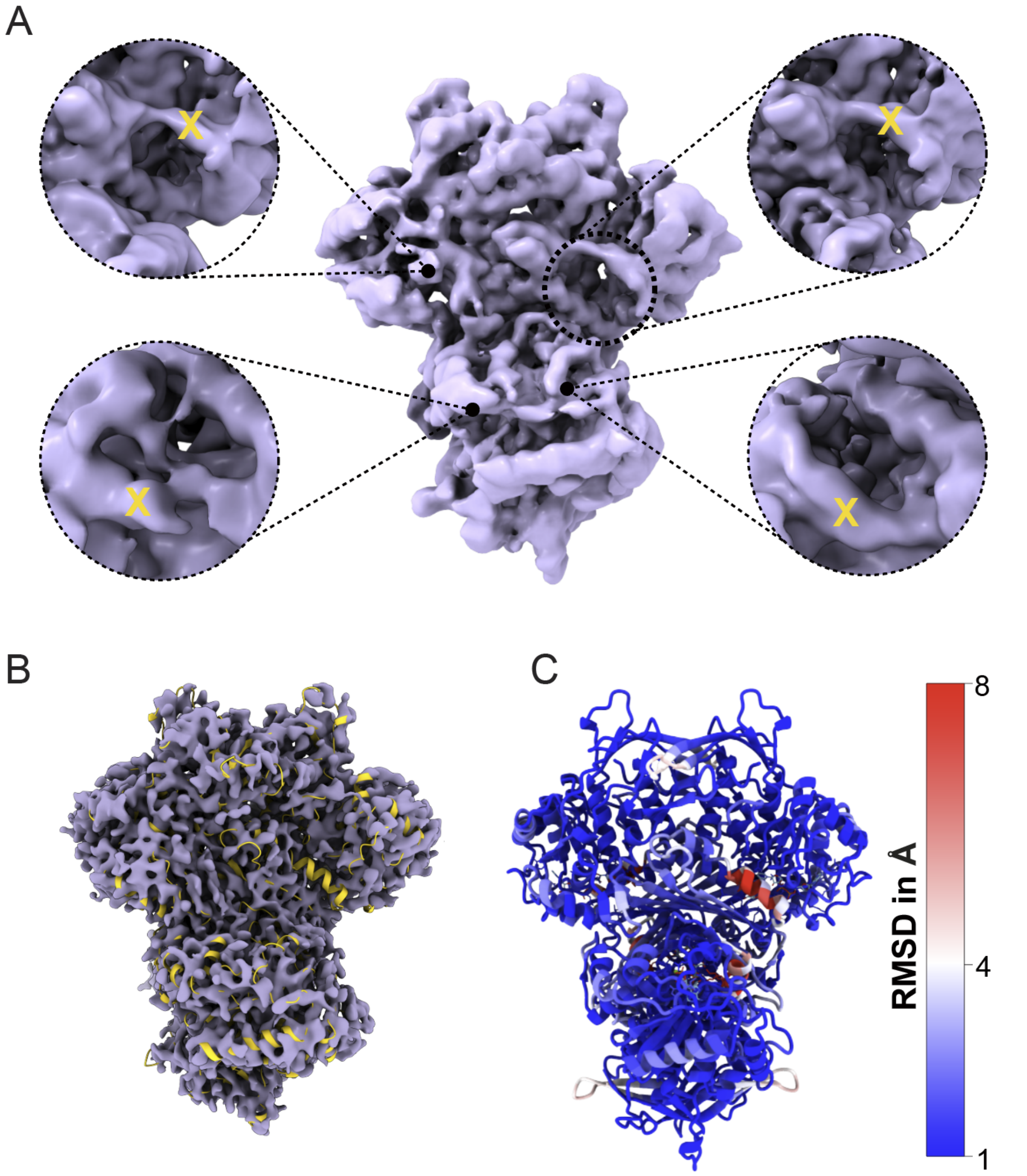

Fig. S17. Active site population across states. All three states identified via 3DVA were analyzed for the existence of the respective cofactors. Active site occlusion is displayed across states, revealing only states 2 and 3 to be available for substrate binding. NAD and a signature relating to the identified ion are present in all states. Only states 1 and 2 showed a density in line with the acyclic intermediates of the isomerization (contour level 0.075).

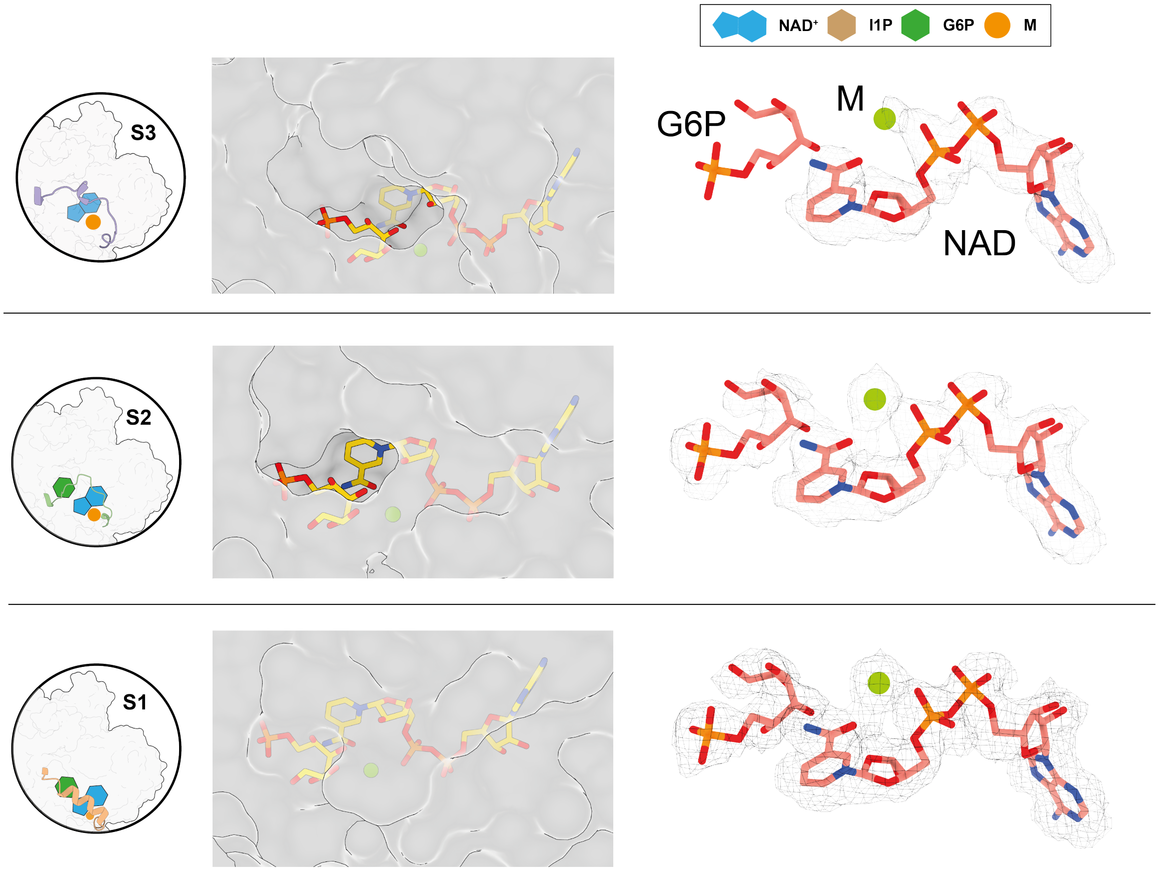

Fig. S18. Conserved PTM sites in MIPS. Previously identified phosphorylation sites (10) were mapped onto the model from *T. thermophila*.

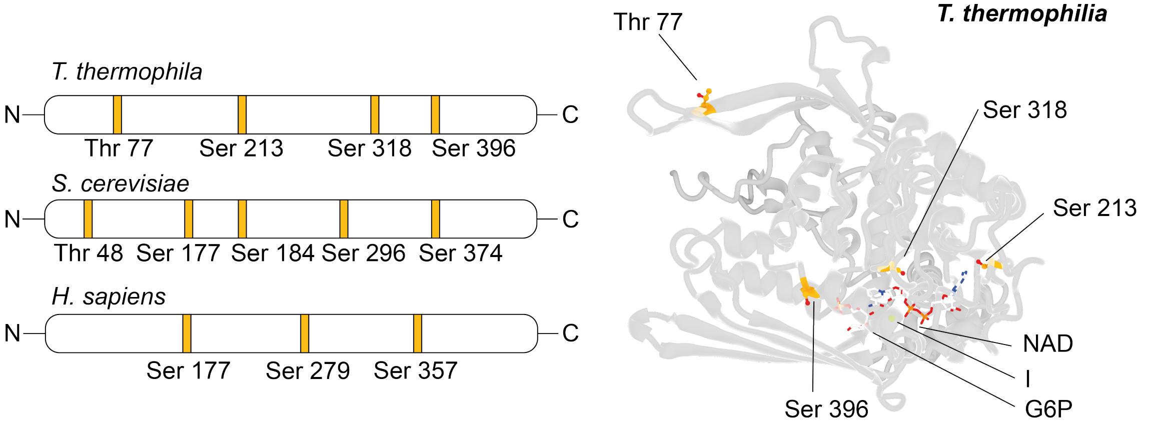

Fig. S19. Phosphate standard calibration curve for activity assay. To determine the concentration of released phosphate a phosphate standard was titrated, exchanging the sample for the respective standard concentration.

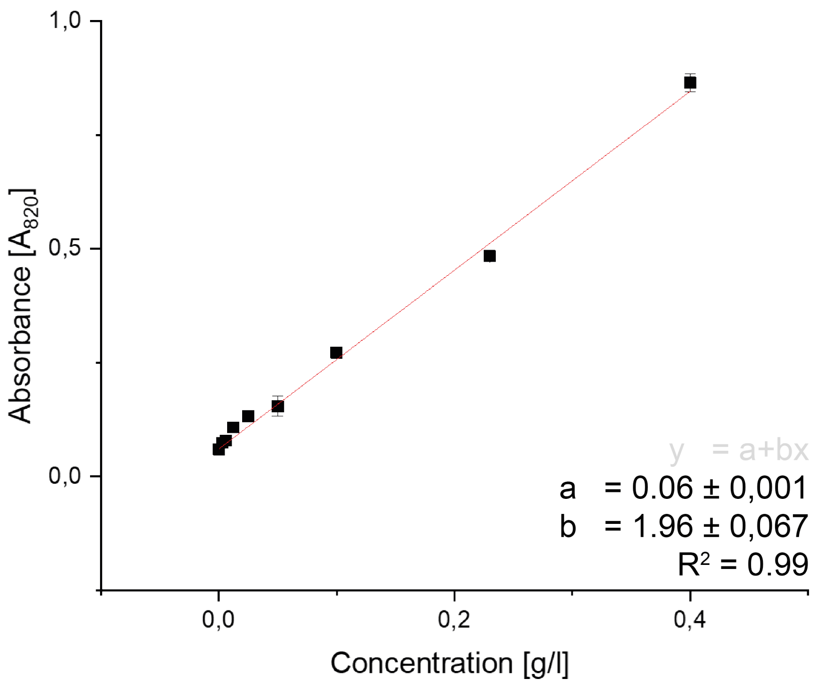

Fig. S20. Energetic evaluation of active site intermediates.

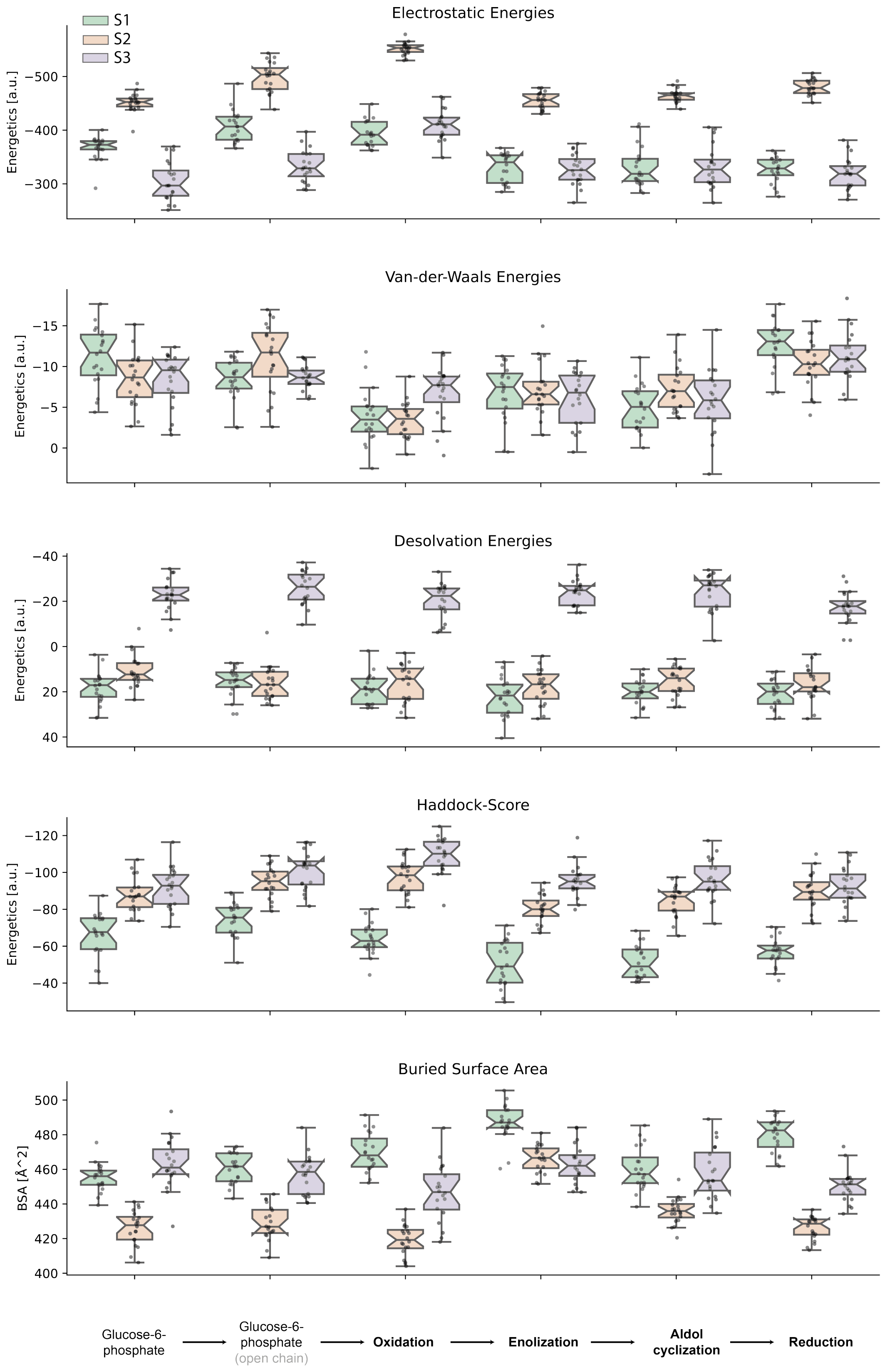

Fig. S21. Sequence alignment of MIPS orthologues. A sequence alignment for MIPS from *T. thermophilia* (G0SDP4), *M. tuberculosis* (P9WKI1), *S. cerevisiae* (P11986), *C. elegans* (G5ED01), *H.Sapiens* (Q9NPH2), *A. thaliana* (Q38862), *H. thetaiotaomicron* (Q8A7J8) and *A. fulgidis* (O28480) using MUSCLE (11) with default settings. Conservation and the consensus sequence are displayed.

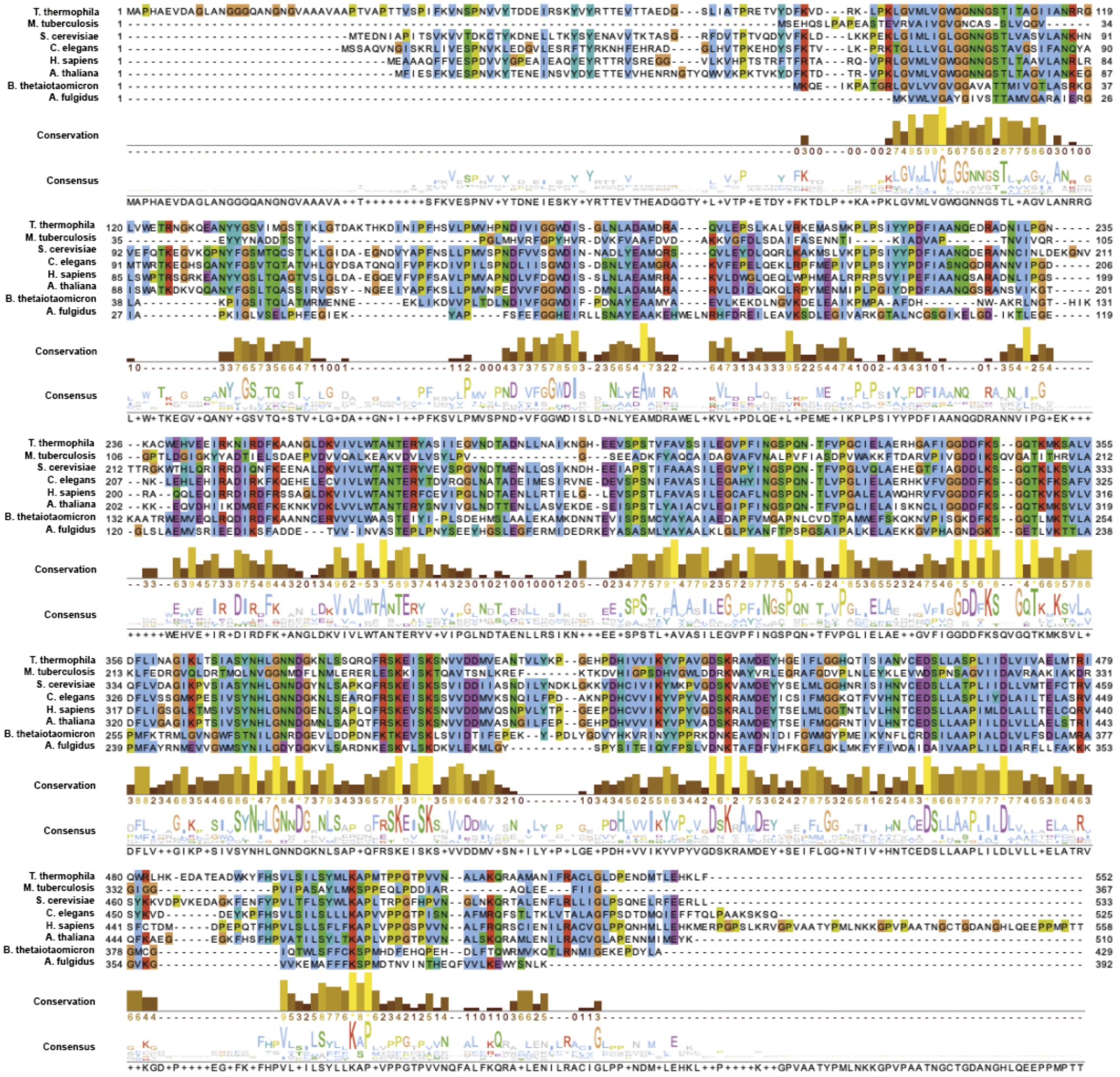

Table S1. Reconstruction and refinement statistics.

	MIPS state 1	MIPS state 3	MIPS state 2
**Data collection and processing**			
Magnification (X)	240.000		
Voltage (kV)	200		
Microscope model	TFS Glacios		
Camera Model	TFS Falcon IIIEC		
Number of frames	13		
Electron exposure (e-/Å^2)	28		
Per-Frame exposure (e-/Å^2)	2.3		
Defocus range (µm)	- 2.5 to - 1		
Pixel size (Å)	0.59		
Images (number aquired)	6261		
Aquisition software	TFS EPU 2		
Symmetry imposed	D2		
Final particle images (number)	255.354	19.408	58.544
Map resolution (Å)	2.48	3.2	2.8
FSC treshold	0.143		
Map B-factor	98.3	69.9	92.2
**Refinement**			
Initial model used	0 (not modified)		
Map sharpening B factor (Å^2)	0 (not modified)		
Model composition			
**Composition (#)**			
Chains	4	1	1
Atoms	3942		
Residues	501		
Ligands	NAD:1<br>0		
Water	0		
**Bonds (RMSD)**			
Length (Å) (# > 4 σ)	0	0	0
Angles (°) (# > 4 σ)	1.1	0	0.767
**MolProbity score**	1.58	1.85	2.08
**Clash score**	9.55	12.77	13.88
**Ramachandran plot (%)**			
Outliers	0		
Allowed	2.15	3.13	2.62
Favoured	97.85	96.87	97.38
**Rama-Z (Ramachandran plot Z-Score, RMSD)**			
whole (N=2060)	-0.09 (0.17)	-1.15 (0.36)	0.15(0.36)
helix (N=748)	-0.22 (0.18)	-1.11 (0.36)	-0,12(0.37)
sheet (N=368)	0.88 (0.26)	- 0.75 (0.48)	1.19(9.53)
loop (N=944)	-0.15 (0.19)	-0.24 (0.43)	-0.04(0.40)
**Rotamer outliers (%)**	1.14	1.14	2.58
**Cβ outlier (%)**	0		
**Peptide plane (%)**			
Cis proline/general	0.0/0.0	0.0/0.0	0
Twisted proline/general	0.0/0.0	0.0/0.0	0
**CaBLAM outliers (%)**	0.98	1.38	1.01
**Model vs. Data**			
CC (mask)	0.83	0.80	0.74
CC (box)	0.65	0.42	0.32
CC (volume)	0.79	0.75	0.74
CC (main chain)	0.80	0.77	0.70
CC (side chain)	0.76	0.75	0.66

Table S2. MIPS structure comparison and HADDOCK refinement (12) of the horizontal interface.

ID	Method	Organism	Expr.	Substrates	Helix	Haddock score		Electr.		Desol.		VdW		BSA	
						x	σ	x	σ	x	σ	x	σ	x	σ
1GR0	XRAY	*M. tub.*	*E.coli*	NAD; CAC;ZN		-286	2	-480	12	-36	5	-154	5	3584	46
1JKF	XRAY	*S. cer.*	*E.coli*	NAD		-305	5	-523	56	-48	10	-154	3	4123	90
1JKI	XRAY	*S. cer.*	*E.coli*	NAI; DG6; NH4		-439	11	-597	65	-66	13	-254	2	6208	41
1LA2	XRAY	*S. cer.*	*E.coli*	NAD, MSE		-469	7	-681	41	-92	4	-240	6	6233	37
1P1F	XRAY	*S. cer.*	*E.coli*	/		-486	7	-612	43	-122	5	-242	4	6361	63
1P1I	XRAY	*S. cer.*	*E.coli*	NAD		-463	7	-574	65	-104	5	-245	3	6285	64
1P1J	XRAY	*S. cer.*	*E.coli*	NAI;PO4; GOL		-431	3	-551	16	-50	3	-271	3	6242	22
1P1K	XRAY	*S. cer.*	*E.coli*	NAI		-425	12	-581	32	-43	6	-266	3	6397	31
1RM0	XRAY	*S. cer.*	*E.coli*	NAI;D6P; Mn		-420	6	-674	37	-22	8	-263	5	6295	29
1U1I	XRAY	*A. ful.*	*E.coli*	NAD; PO4;K		-470	3	-768	43	-146	2	-171	8	4902	56
1VKO	XRAY	*C.ele.*	*E.coli*	NAD; POP;IOD;K;CL		-430	11	-1346	78	42	4	-203	10	6232	56
7NWR	XRAY	*B. the.*	*E.coli*	NAD;NA		-286	2	-779	26	-63	7	-163	3	3877	12
	Cryo	*T. the.*	*native*	NAD		**-384.**	**3.7**	**-1157**	**41**	**56**	**8**	**-209**	**5**	**6323**	**38**
	AF2	*H. sap.*				-517	3	-1238	21	-60	4	-210	3	6342	45

Table S3. MIPS structure comparison and HADDOCK (12) refinement of the vertical interface.

ID	Method	Organism	Expr.	Substrates	Helix	Score		Electr.		Desolv.		VdW		BSA	
						x	σ.	x	σ.	x	σ.	x	σ.	x	σ.
1GR0	XRAY	*M. tub.*	*E.coli*	NAD; CAC; ZN		-509	5	-676	17	-59	6	-315	2	7912	28
1JKF	XRAY	*S. cer.*	*E.coli*	NAD		-1704	1	-2339	61	-239	9,	-998	10	24131	68
1JKI	XRAY	*S. cer.*	*E.coli*	NAI; DG6; NH4		-1763	5	-2104,	66	-249	8	-1094	10	26107	41
1LA2	XRAY	*S. cer.*	*E.coli*	NAD, MSE		-1849	6	-2573	53	-258	4	-1077	8	26415	85
1P1F	XRAY	*S. cer.*	*E.coli*			-1875	20	-2340	43	-295	22	-1113	6	26467	78
1P1I	XRAY	*S. cer.*	*E.coli*	NAD		-1921	8	-2679	53	-286	6	-1100	13	26280	153
1P1J	XRAY	*S. cer.*	*E.coli*	NAI;PO4; GOL		-1782	8	-2260	45	-210	6	-1120	8	26043	89
1P1K	XRAY	*S. cer.*	*E.coli*	NAI		-1806	5	-2172	52	-234	2	-1139	9	26595	79
1RM0	XRAY	*S. cer.*	*E.coli*	NAI;D6P; Mn		-1762	4	-2205	38	-178	16	-1143	14	26419	125
1U1I	XRAY	*A. ful.*	*E.coli*	NAD; PO4; K		-866	6	-799	30	-226	12	-481	4	10607	48
1VKO	XRAY	*C.ele.*	*E.coli*	NAD; POP; IOD; K;CL		-2028	7	-3038	53	-265	4	-1156	12	27009	117
7NWR	XRAY	*B. the.*	*E.coli*	NAD; NA		-1069	8	-1220	7	-224	8	-601	8	13355	65
	Cryo	*T. the.*	*native*	NAD		-1523	**7**	**-1874.50**	**108**	**-173**	**10.20**	**-975**	**8**	**23739**	**130**
	AF 2	*H. sap.*				-1917	6	-3575	45	-144	8	-1058	9	27093	39

Table S4. MIPS activity assay. Background subtracted absorbance (A_{820}), as well as the normalized abundance and the concentration of phosphate released by G6P conversion (c_P) is shown.

Fr.	Rep. 1			Rep. 2			Rep. 3		
	A_{820}	Norm. Abund.	c_P [g/l]	A_{820}	Norm. Abund.	c_P [g/l]	A_{820}	Norm. Abund.	c_P [g/l]
**30**	0,000	6,74218E-4	/	0,014	0,034	/	0,015	0,128	/
**31**	0,003	0,00674	/	0,053	0,130	/	0,035	0,308	/
**32**	0,02875	0,07754	/	0,251	0,623	0,097	0,064	0,559	0,001
**33**	0,13785	0,37176	0,03935	0,403	1	0,174	0,082	0,718	0,011
**34**	0,3708	1	0,158	0,401	0,995	0,173	0,107	0,938	0,023
**35**	0,3627	0,97816	0,15388	0,371	0,921	0,158	0,114	1	0,027
**36**	0,21125	0,56971	0,07674	0,269	0,668	0,106	0,080	0,700	0,010
**37**	0,0977	0,26348	0,0189	0,179	0,445	0,060	0,079	0,692	0,009
**38**	0,0155	0,0418	/	0,088	0,219	0,014	0,023	0,198	/
**39**	0,0214	0,05771	/	0,064	0,158	0,002	0,059	0,520	/
**40**	0,0028	0,00755	/	0,070	0,175	0,005	0,051	0,449	/

Table S5. Identified folding events. To assess how active site capping is preserved through a transition from disorder to order, we examined isomerases listed in the Protein Data Bank (PDB) (13), pinpointing folding processes associated with the functionality of the active site. Out of 344 instances, representing those with over three structures submitted to the PDB, we detected 30 instances of folding events related to the enzyme's activity.

Uniprot Entry	PDB	EC number	Enzyme description
Q9I7C2	6M1J 6M1S 7PTF 7PTG 8BN6	5.6.2.2	DNA gyrase subunit B
P32170	1D8W 1DE5 1DE6	5.3.1.14	L-rhamnose isomerase
P60340	1K8W 1R3F 1ZL3	5.4.99.25	tRNA pseudouridine synthase B
P56839	1M1B 1PYM 1S2T 1S2U 1S2V 1S2W	5.4.2.9	Phosphoenolpyruvate phosphomutase
Q56320	1DL3 1LBM 1NSJ	5.3.1.24	N-(5'-phosphoribosyl)anthranilate isomerase
Q8GIQ0	1NXM 1NYW 1NZC 2IXL	5.1.3.13	dTDP-4-dehydrorhamnose 3,5-epimerase
P14565	1P4D 2A0I 2L8B 2Q7T 2Q7U 3FLD 5N8O	5.6.2.1	Multifunctional conjugation protein TraI
P16250	1VZW 2VEP 2X30 5DN1	5.3.1.24	Phosphoribosyl isomerase A (yeast)
P9WMM5	2Y85 2Y88 2Y89 3ZS4	5.3.1.24	Phosphoribosyl isomerase A (myco)
Q13907	2DHO 2I6K 2ICJ 2ICK	5.3.3.2	Isopentenyl-diphosphate Delta-isomerase 1
P9WFX1	2G5F 2I6Y 3LOG 3RV6 3RV7 3RV8 3RV9 3ST6 3VEH 6ZA4 6ZA5 6ZA6	5.4.4.2	Salicylate synthase
Q4K9X1	3CT2 3DGB 3FJ4	5.5.1.1	Muconate cycloisomerase
A1B198	4E8G 4IZG 4J1O	5.1.1.22	4-hydroxyproline betaine 2-epimerase
P9WIQ1	1V0J 4RPG 4RPH 4RPJ 4RPK 4RPL	5.4.99.9	UDP-galactopyranose mutase
Q38802	3PYA 3PYB 4LIX	5.5.1.13	Ent-copalyl diphosphate synthase, chloroplastic
Q9Y606	4IQM 4ITS 4J37 4NZ6 4NZ7	5.4.99.12	Pseudouridylate synthase 1 homolog
V9P0A9	4C9S 4C9T 4D06 4D4F 8B7R 8B7U 8B7Z	5.5.1.6	Chalcone isomerase
P10372	5A5W 5AB3 5ABT 5AC6 5AC7 5AC8 5AHE 5AHF 5AHI 5G1T 5G1Y 5G2H 5G2I 5G2W 5G4E 5G4W 5G5I 5L6U 5L9F	5.3.1.16	1-(5-phosphoribosyl)-5-[(5-phosphoribosylamino)methylideneamino] imidazole-4-carboxamide isomerase
P34808	5WC0 5WC1 5WCB 6B5D 6UGD 6UGE 6UGF	5.6.1.1	Meiotic spindle formation protein mei-1
Q6NER4	4XGK 5BR7 5EQF 6D2E 6D2G	5.4.99.9	UDP-galactopyranose mutase
Q9GZT4	3L6B 3L6R 5X2L 6SLH 6ZSP 6ZUJ 7NBC 7NBD 7NBF 7NBG 7NBH	5.1.1.18	Serine racemase
A0A0H3JU78	6JIS 6JIW 6L4L	5.1.1.24	Histidine racemase
A0QNE0	4B6C 4BAE 6ZT3 6ZT5	5.6.2.2	DNA gyrase subunit B
A0A348FUE1	6V0K 7S09 7S0A 7S0H 7S0L 7S0M	2.5.1.29	Copalyl diphosphate synthase
A0A7Z2BHK4	7PQI 7PQL 7PQM	5.6.2.2	DNA gyrase subunit B
A4XGA6	4Z4J 4Z4L 7D5G	5.1.3.11	Cellobiose 2-epimerase
K7QVW7	5BTU 5BU3 7DVK	/	Spiro-conjugate synthase
P09095	5N81 5N82 7YWJ 7YWK	5.1.1.11	Tyrocidine synthase 1
Q0P8I4	7AN4 7ANG 7M14 7M15	5.1.3.13	dTDP-4-dehydrorhamnose 3,5-epimerase

SI References

1. S. Amlacher *et al.*, Insight into structure and assembly of the nuclear pore complex by utilizing the genome of a eukaryotic thermophile. *Cell* **146**, 277-289 (2011).
2. F. L. Kyrilis *et al.*, Integrative structure of a 10-megadalton eukaryotic pyruvate dehydrogenase complex from native cell extracts. *Cell reports* **34**, 108727 (2021).
3. D. Szklarczyk *et al.*, STRING v11: protein-protein association networks with increased coverage, supporting functional discovery in genome-wide experimental datasets. *Nucleic Acids Research* **47**, D607-D613 (2019).
4. Y. Z. Tan *et al.*, Addressing preferred specimen orientation in single-particle cryo-EM through tilting. *Nat Methods* **14**, 793-796 (2017).
5. J. Jumper *et al.*, Highly accurate protein structure prediction with AlphaFold. *Nature* **596**, 583-589 (2021).
6. A. J. Stein, J. H. Geiger, The crystal structure and mechanism of 1-L-myo-inositol- 1-phosphate synthase. *The Journal of biological chemistry* **277**, 9484–9491 (2002).
7. M. Varadi *et al.*, AlphaFold Protein Structure Database: massively expanding the structural coverage of protein-sequence space with high-accuracy models. *Nucleic Acids Research* **50**, D439-D444 (2022).
8. G. Pintilie *et al.*, Measurement of atom resolvability in cryo-EM maps with Q-scores. *Nature methods* **17**, 328–334 (2020).
9. A. Punjani, D. J. Fleet, 3D variability analysis: Resolving continuous flexibility and discrete heterogeneity from single particle cryo-EM. *Journal of structural biology* **213**, 107702 (2021).
10. R. M. Deranieh, Q. He, J. A. Caruso, M. L. Greenberg, Phosphorylation regulates myo-inositol-3-phosphate synthase: a novel regulatory mechanism of inositol biosynthesis. *The Journal of biological chemistry* **288**, 26822–26833 (2013).
11. R. C. Edgar, MUSCLE: multiple sequence alignment with high accuracy and high throughput. *Nucleic Acids Research* **32**, 1792–1797 (2004).
12. G. C. P. van Zundert *et al.*, The HADDOCK2.2 Web Server: User-Friendly Integrative Modeling of Biomolecular Complexes. *Journal of molecular biology* **428**, 720–725 (2016).
13. H. M. Berman *et al.*, The Protein Data Bank. *Nucleic Acids Research* **28**, 235–242 (2000).

Dataset S1 (separate file). MS analysis of the soluble, fractionated proteome of *Thermochaetoides thermophila.*
